# Generative AI enhances individual creativity but reduces the collective diversity of novel content

**DOI:** 10.1126/sciadv.adn5290

**Published:** 2024-07-12

**Authors:** Anil R. Doshi, Oliver P. Hauser

**Affiliations:** ^1^ Department of Strategy and Entrepreneurship, UCL School of Management, London, UK.; ^2^ Department of Economics, University of Exeter, Exeter, UK.; ^3^ Institute for Data Science and Artificial Intelligence, University of Exeter, Exeter, UK.

## Abstract

Creativity is core to being human. Generative artificial intelligence (AI)—including powerful large language models (LLMs)—holds promise for humans to be more creative by offering new ideas, or less creative by anchoring on generative AI ideas. We study the causal impact of generative AI ideas on the production of short stories in an online experiment where some writers obtained story ideas from an LLM. We find that access to generative AI ideas causes stories to be evaluated as more creative, better written, and more enjoyable, especially among less creative writers. However, generative AI–enabled stories are more similar to each other than stories by humans alone. These results point to an increase in individual creativity at the risk of losing collective novelty. This dynamic resembles a social dilemma: With generative AI, writers are individually better off, but collectively a narrower scope of novel content is produced. Our results have implications for researchers, policy-makers, and practitioners interested in bolstering creativity.

## Teaser

Generative AI can enhance the creativity of short stories but may limit the variation in diverse outputs.

## INTRODUCTION

Creativity is fundamental to innovation and human expression through literature, art, and music (*1*). However, the emergence of generative artificial intelligence (AI) technologies—such as large language models (LLMs) as used in our study—is challenging several long-standing assumptions about the uniqueness and superiority of human-generated content (*2*). Generative AI is able to create new content in text (e.g., ChatGPT), images (e.g., Midjourney), audio (e.g., Jukebox), and video (e.g., Pictory). While generative AI has previously been shown to enable joint AI-human storyline development (*3*), increase quality and efficiency of production of typical white-collar work (*4*), promote productivity in customer support relations (*5*, *6*), speed up programming tasks (*7*), and enhance persuasion messaging (*8*), little is known about generative AI’s potential impact on a fundamental human behavior: the ability of humans to be creative.

Taking a first step toward understanding the relationship between generative AI and human creativity, we focus specifically on the role of generative AI on affecting creative output through the expression of short (or micro) fiction. While creating written output is only one form of human expression, its use is widespread across the economy (e.g., business plans, sales pitches, or marketing campaigns) and society (e.g., books and social media). Here, we study how generative AI affects participants’ ability to produce this particular type of creative written output (*9*). While we did not introduce financial incentives for performance or creativity [as they have previously led to mixed results (*10*)], we provided guidance to authors to write a story on a randomly assigned topic and gave instructions on the length of the story and the target audience.

Creativity is typically assessed across two dimensions: novelty and usefulness (*11*, *12*). Because the two were designed for other creativity tasks [such as idea generation, see (*13*), or physical design task, see (*11*)], we slightly adjusted some components of the constructs. Novelty assesses the extent to which an idea departs from the status quo or expectations. In our study, following the previous literature, the novelty index captured the story’s novelty, originality, and rarity. Usefulness reflects the practicality and relevance of an idea, which we interpret as the possibility that this short story could become a publishable product, such as a book, if developed further: Therefore, our usefulness index was adjusted to capture the story’s appropriateness for the targeted audience, feasibility of being developed into a complete book, and likelihood of a publisher developing the book.

There are at least two ways in which the availability of generative AI can affect creative writing in this context. On the one hand, generative AI may enhance: Generated ideas from AI may be used as a “springboard” for the human mind, providing potential starting points that can result in a “tree structure” of different storylines (*3*, *14*). It can also offer multiple starting venues that help a human writer overcome “writer’s block” and the fear of a blank page (*15*). If this is the case, we would expect generative AI to lead to more creative written output generated by human writers.

Conversely, generative AI may hamper: By anchoring the writer to a specific idea, or starting point for a story, generative AI may restrict the variability of a writer’s own ideas from the start, inhibiting the extent of creative writing. Moreover, the output offered by generative AI may be derivative and thus not provide a fertile ground for new and creative ideas. If this is the case, we would expect generative AI to lead to more similar stories and potentially less creative written output generated by human writers. Note that these two pathways in which generative AI can affect creative writing may not be mutually exclusive: It is possible that generative AI enhances a human’s ability to write creative stories in some ways (e.g., novelty) but not in others (e.g., usefulness) (*12*).

**Fig. 1.**
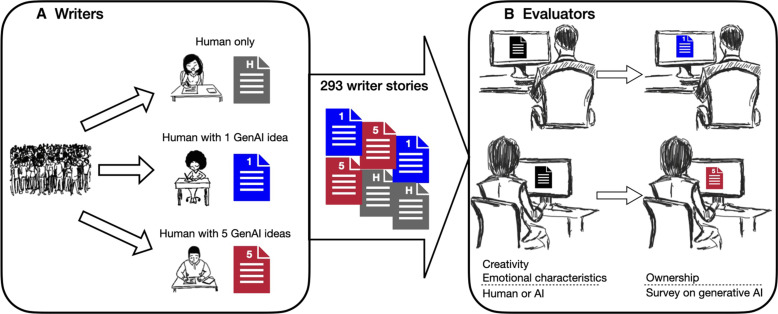
Visual representation of experimental design. (**A**) Participants are recruited, provide consent to participate in the study, and complete the divergent association task (DAT)—a measure of an individual’s inherent creativity (*25*)—before being randomly assigned to one of three experimental conditions: a Human-only condition where the story was written with no generative AI assistance, a Human with one GenAI idea condition, and a Human with five GenAI ideas condition. A total of 293 stories are collected and then passed to evaluators. (**B**) Evaluators provide ratings on six randomly assigned stories. The evaluators cycle through each story three times. First, before any information revelation, the evaluator assesses the creativity and emotional characteristics of the story. Second, the evaluator is asked to assess how likely the story was written by an AI versus a human. Third, the evaluator is told about whether the writer had access to and used generative AI and then provides responses about the ownership claim of the writer of each story. Evaluators then provide general responses to their views of generative AI.

This paper aims to provide an initial answer to these questions through a preregistered, two-phase experimental online study on written creative output (see Fig. 1 for the experimental design and Materials and Methods for details) (*16*). In the first phase of our study, we recruited a group of *N* = 293 participants (“writers”) who are asked to write a short, eight-sentence story that is “appropriate for a teenage and young adult audience,” and we indicate to writers, “You can write about anything you like.” [We drew inspiration from the emergence of the “micro” genre in creative outputs, including “microfiction” (*17*) and “micro-videos” (*18*) where creativity emerges amidst brevity; indeed, the famous “six-word story” often attributed to Ernest Hemingway highlights the creative power of a concise plot (*19*).] Participants were randomly assigned to one of three conditions: Human-only, Human with one GenAI idea condition, and Human with five GenAI ideas (see table S1 for balance across conditions).

In our Human-only baseline condition, writers were assigned the task with no mention of or access to generative AI. In the two generative AI conditions, we gave writers the option to call upon a generative AI platform (OpenAI’s GPT-4 LLM) to provide a three-sentence starting idea to inspire their own story writing. In one of the two generative AI conditions (Human with five GenAI ideas), writers could choose to receive up to five generative AI ideas, each providing a possibly different inspiration for their story. After completing their story, writers were asked to self-evaluate their story on novelty, usefulness, and several emotional characteristics (see section S1 for all study questions).

In the second phase, the stories composed by the writers were evaluated by a separate group of *N* = 600 participants (“evaluators”) (see table S2 for balance across conditions). Evaluators read six randomly selected stories without being informed about writers being randomly assigned to access generative AI in some conditions (or not). All stories were evaluated by multiple evaluators on novelty, usefulness, and several emotional characteristics, which comprise key outcome variables related to our main research question (see section S1).

For exploratory purposes, additional questions not directly related to our main research question were included after the main outcome variables. Specifically, after disclosing to evaluators whether generative AI was used during the creative process (*20*), we asked evaluators to rate the extent to which ownership and hypothetical profits should be split between the writer and the AI (*21*). We also elicited evaluators’ general views on the extent to which they believe that the use of AI in producing creative output is ethical, how story ownership and hypothetical profits should be shared between AI creators and human creators, and how AI should be credited in the involvement of the creative output (*22*, *23*). The results of these exploratory analyses are included in section S5.

## RESULTS

### Baseline versus generative AI conditions

As part of our preregistration, we tested whether the baseline Human-only condition differed from the combined generative AI conditions. We find that generative AI assistance increases both the novelty and usefulness of stories (results are discussed in section S4). To better understand how greater availability of generative AI ideas affects the enhancement in creativity, we follow our preregistration to estimate the causal impact of the two generative AI conditions separately. Writers in the Human with one GenAI idea condition are given the choice to request a single generative AI story idea, while writers in the Human with five GenAI ideas condition are given the option to access up to five generative AI story ideas.

Across the two generative AI conditions, 88.4% of participants chose to call upon generative AI at least once to provide an initial story idea. Of the 100 writers in the Human with one GenAI idea condition, 82 opted to generate one, while 93 of 98 writers in the Human with five GenAI ideas condition did so. When given the option to call upon generative AI more than once in the Human with five GenAI ideas condition, participants did so on average 2.55 times, with 24.5% requesting the maximum of five generative AI ideas.

**Fig. 2.**
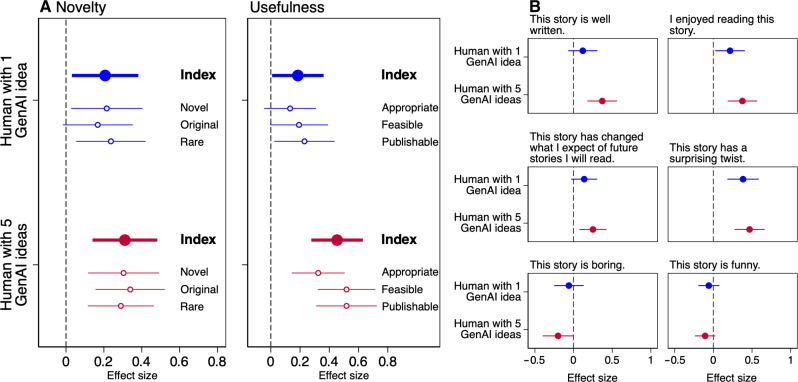
Evaluation of creativity and emotional characteristics by third-party evaluators. (**A**) Compares novelty and usefulness indices (with constituent components of each index below) of participants in the Human-only condition (dashed vertical line) to participants who had access to one generative AI idea (top half in each panel, blue) or five generative AI ideas (bottom half, red). (**B**) Compares emotional characteristics of the Human-only condition (dashed vertical line) to Human with one GenAI idea and Human with five GenAI ideas conditions.

We find that, while having access to one generative AI idea leads to somewhat greater creativity, the most gains (and statistically significant differences in our preregistered indices) come from writers who have access to five generative AI ideas (Fig. 2A; fig. S1 shows a violin plot of raw data). With respect to novelty, writers in the Human with one GenAI idea condition experience an increase of 5.4% (*b* = 0.207, *P* = 0.021, see table S3) over writers without generative AI access, whereas writers in the Human with five GenAI ideas condition show an increase in novelty of 8.1% (*b* = 0.311, *P* < 0.001) over writers without generative AI access.

The results of story usefulness are even more notable. The usefulness of stories from writers with access to one generative AI idea is 3.7% (*b* = 0.185, *P* = 0.039) higher than that of writers with no generative AI access. Having access to up to five AI ideas increases usefulness by 9.0% (*b* = 0.453, *P* < 0.001) over those with no generative AI access and 5.1% (*P* = 0.0012, compared to the Human with one GenAI idea condition mean of 5.21) over those with access to one generative AI idea. The overall results suggest that having access to more AI ideas lead to more creative storytelling. The novelty and usefulness index results are qualitatively unchanged when we include evaluator fixed effects, story order fixed effects, story topic fixed effects, and an indicator variable that equals one if the writer accessed at least one generative AI idea (see table S4).

In contrast, writers self-assessing their own stories show no statistically significant differences in the novelty and usefulness between authors who were offered generative AI ideas and those who were not (see table S5).

### Exploratory analyses: Emotional characteristics

Next, we turn to measures that gauge the evaluators’ emotional responses to the stories, based on categories of general reader interest, including how well written, enjoyable, funny, and boring the stories are and the extent to which the story has a plot twist. We also asked whether the story changed the reader’s expectations about future stories [based on literature theorist Robert Jauss’ conception of more novel literature changing the reader’s “horizon of expectations” in the future (*24*)].

As illustrated in Fig. 2B, we find that stories written by writers with access to generative AI ideas are more enjoyable (Human with one GenAI idea condition: *b* = 0.216, *P* = 0.028; Human with five GenAI ideas condition: *b* = 0.375, *P* < 0.001, see table S6) and are more likely to have plot twists (Human with one GenAI idea condition: *b* = 0.384, *P* < 0.001; Human with five GenAI ideas condition: *b* = 0.468, *P* < 0.001). Relative to Human-only stories, when the writer had access to up to five generative AI ideas, the stories are considered to be better written (*b* = 0.372, *P* < 0.001), have more of an effect on the evaluator’s expectations of future stories (*b* = 0.251, *P* = 0.005), and be less boring (*b* = −0.200, *P* = 0.049). Stories in the Human with five GenAI ideas condition are, however, not evaluated as more funny than those in the Human-only condition; if anything, the coefficient is negative but not statistically significant (*b* = −0.106, *P* = 0.115).

Again, writers’ self-assessments of their own stories show no statistically significant differences in the story characteristics across conditions (see table S7).

### Heterogeneity by inherent creativity

Because our human writers were not specifically selected for their creative predispositions or work in creative industries, we are able to take advantage of natural variation in the underlying creativity of writers in our sample. To do so, we had writers complete a divergent association task (DAT) before writing their stories (*25*). The task entails providing 10 words that are as different from each other as possible. The DAT score is the cosine distance of the underlying word embeddings (scaled to 100) and captures the individual’s inherent creativity. In our sample, the DAT score had a mean of 77.24 and an SD of 6.48. The computation of DAT requires 7 of 10 submitted terms to be valid (i.e., single words that appear in the dictionary). Two writers failed to properly submit seven valid words; thus, the DAT score was successfully computed for 291 of 293 writers.

First, we look at whether different writers engaged with generative AI more than others: We do not find differences between more creative writers and less creative writers in how frequently they accessed generative AI ideas in the two generative AI conditions (see table S8). Among both more and less creative writers in the Human with five GenAI ideas condition, all five ideas were requested 24.5% of the time. In short, we do not observe any differences in how generative AI was accessed based on the inherent creativity of the writer.

**Fig. 3.**
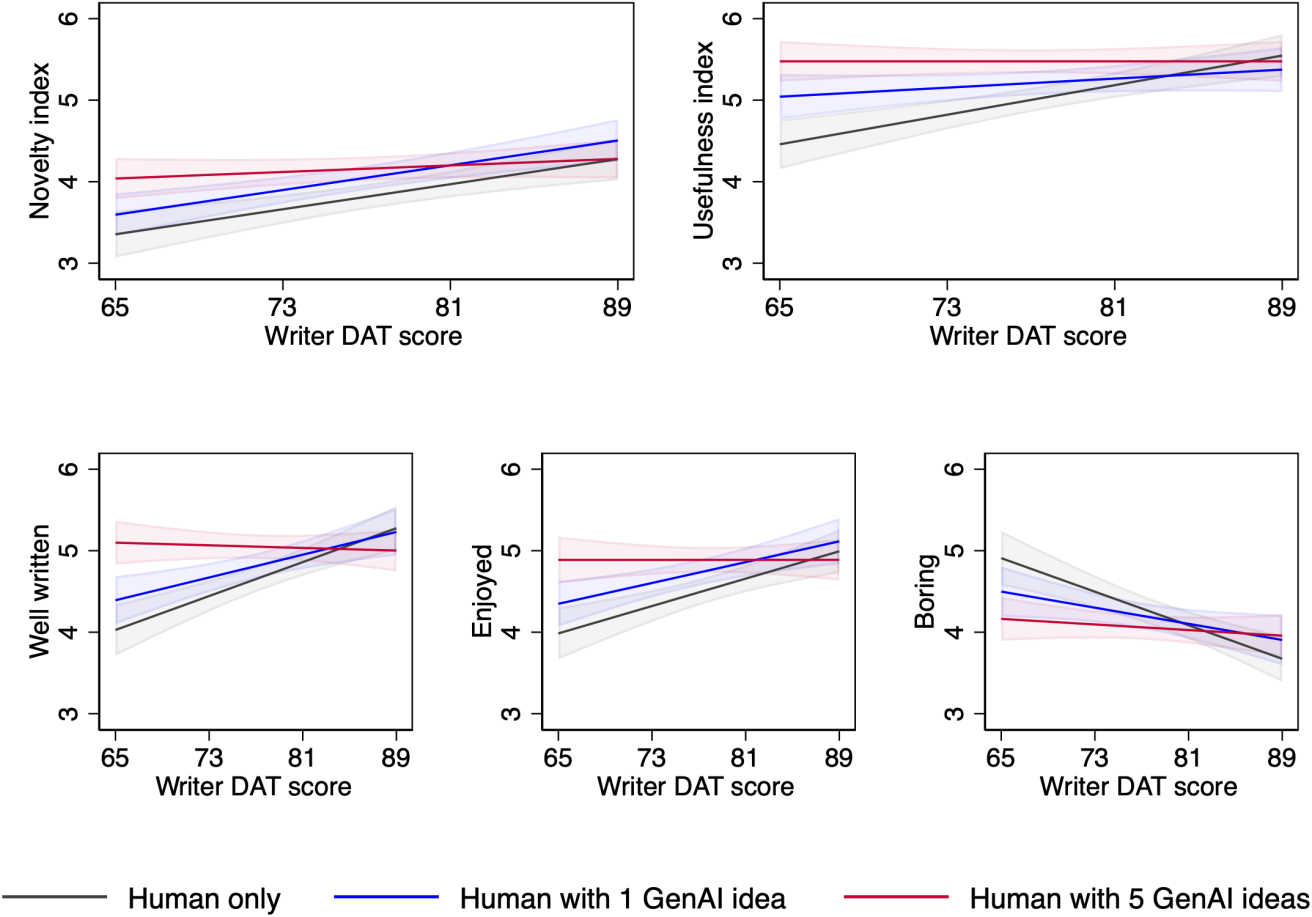
Marginal effect of writer’s inherent creativity (as measured by DAT score) on the creativity indices and on select emotional characteristics by condition.

Next, we interact the continuous DAT score with our conditions (see tables S9 and S10 for results on all outcome variables). Figure 3 presents graphs that show the differential effect of generative AI ideas on select variables, based on the inherent creativity of the writer (see fig. S2 for graphs of the remaining outcome variables). Among the most inherently creative writers (i.e., high-DAT writers), there is little effect of having access to generative AI ideas on the creativity of their stories. Across all conditions, high-DAT writers’ stories are evaluated relatively highly, in terms of both novelty and usefulness, and providing them with access to generative AI does not affect their high evaluations. We observe a similar result among high-DAT writers for how well the story was written, how enjoyable, and, conversely, how boring it is: Having access to generative AI does not affect high-DAT writers’ already good performance on these outcomes.

In contrast, access to generative AI ideas substantially improves the creativity and select emotional characteristics of stories written by inherently less creative writers (i.e., low-DAT writers). Among low-DAT writers, having access to one generative AI idea improves a story’s novelty by 6.3% and having access to five generative AI ideas yields improvements of 10.7%. Similarly, writers with access to one and five generative AI ideas produce stories that are evaluated more highly on usefulness by 5.5 and 11.5%, respectively. Similar improvements exist for certain story characteristics. For low-DAT writers in the Human with five GenAI ideas condition, assessments of how well the story was written increase by up to 26.6%, enjoyment of the story increases by up to 22.6%, and how boring the story is decreases by up to 15.2%. These improvements in the creativity of low-DAT writers’ stories put them on par with high-DAT writers. In short, the Human with five GenAI ideas condition effectively equalizes the creativity scores across less and more creative writers.

### Similarity of stories

**Fig. 4.**
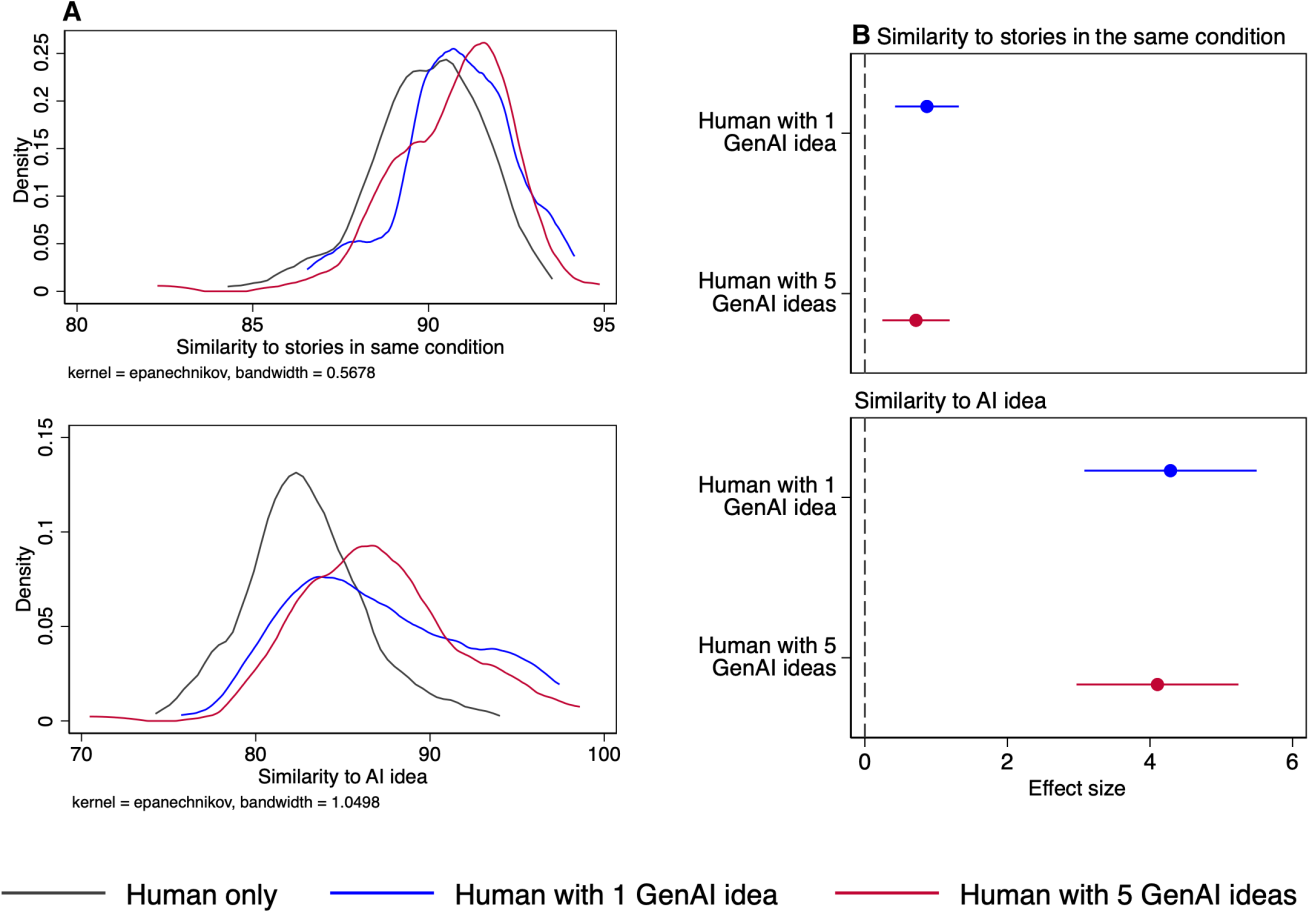
Comparison of similarity of writer stories to generative AI ideas and others stories. (**A**) Kernel density plots comparing story similarity to all other stories in the same condition and ideas produced by generative AI for each condition. (**B**) Compares story outcomes of Human-only (reference category) to humans with access to one and five generative AI ideas.

Thus far, we have focused on the subjective evaluation of third-party readers; now, we turn to a more objective measure of the stories’ content, to understand how generative AI affects the final stories produced. Using embeddings (*26*) obtained from OpenAI’s embeddings application programming interface (API), we were able to compute the cosine similarity of the stories to all other stories within condition as well as the generative AI ideas (Fig. 4). We multiply the cosine similarity score by 100 to arrive at a measure that ranges from 0 to 100.

We look at the similarity of any one story to the “mass” of all stories within the same condition by computing the cosine similarity of the embedding of the focal story with the average embedding of all other stories in the same condition. Our results show that having access to generative AI ideas makes a story more similar to the average of other stories within the same condition (Human with one GenAI idea condition: *b* = 0.871, *P* < 0.001; Human with five GenAI ideas condition: *b* = 0.718, *P* = 0.003, see table S11). To put these values in context, consider that in the Human-only condition, the similarity scores span a range of 8.10 points; therefore, the increase in similarity from having access to one or five generative AI ideas represents 10.7% and 8.9% of the total range, respectively.

To understand why generative AI-inspired stories look more similar to each other, it is instructive to take a closer look at the relationship between generative AI ideas and the stories produced. We compare the cosine similarity of the story embedding to that of the generative AI idea. For stories in the Human-only condition or in one of the generative AI idea conditions where the writer chose not to generate an idea, we randomly assigned a generative AI idea from the pool of ideas (that were created for other writers) within the same story topic. For writers in the two generative AI idea conditions who used the generative AI idea, we selected the first available idea. Then, we tested how similar the stories were to the generative AI ideas. Relative to Human-only, writers in the Human with one GenAI idea condition and Human with five GenAI ideas conditions wrote stories that were 5.2% (*b* = 4.29, *P* < 0.001; compared to a Human-only mean of 82.85) and 5.0% (*b* = 4.11, *P* < 0.001) more similar to the generative AI ideas, respectively. In short, writers in the two generative AI conditions are anchored to some extent on the generative AI idea presented to them.

## DISCUSSION

Generative AI has the potential to markedly affect most aspects of the economy and society at large (*27*, *28*). Previous empirical work has focused on its effects on productivity, routine tasks, sales, resume writing, AI-driven policy design, and joint collaboration between humans and AI, including for scientific and medical tasks (*3*–*6*, *29*–*33*), all of which contribute to our understanding of the potentially transformative impact of generative AI. Here, we extend this work by taking a first step in the direction of studying a question fundamental to all human behavior, which is of both economic and purely expressive value: How does generative AI affect human creativity?

Our work provides a first step toward an answer to this far-reaching question by experimentally studying the causal effect of having access to generative AI on writing short (micro) stories in an online experiment. We find that having access to generative AI causally increases the average novelty and usefulness—two frequently studied dimensions of creativity—relative to human writers on their own. This is driven, in particular, by our experimental condition that enables writers to request multiple generative AI ideas—up to five in our study—each presenting a different starting point, leading to a “tree” branching off to potential storylines (*3*).

Our results provide insight into how generative AI enhances creativity. Having access to generative AI “professionalizes” the stories beyond what writers might have otherwise accomplished alone. The overall effect is a more novel and even more useful story that is well written and enjoyable. However, the gains from writing more creative stories benefit some more than others: Less creative writers experience greater uplifts for their stories, seeing increases of 10 to 11% for creativity and of 22 to 26% for how enjoyable and well written the story is.

We note three additional observations about our findings. First, having access to generative AI effectively equalizes the evaluations of stories, removing any disadvantage or advantage based on the writers’ inherent creativity (*25*). That generative AI particularly benefited less able writers is paralleled in recent studies focusing on other domains in which generative AI has been shown to help less productive workers (*4*, *5*). Second, one might ask whether the generative AI ideas can push the upper bound of creativity of produced stories, beyond what particularly creative humans are capable of on their own. We do not find evidence of this possibility in this study.

Third, after evaluators assessed the stories, we disclosed to them whether the writer received generative AI ideas and what those ideas were. We collected a range of additional (exploratory) outcomes that are not directly related to our primary (preregistered) research questions and therefore not included in the main text, but which we briefly discuss here to inspire future directions of research (see section S5 for details). We find that evaluators imposed an ownership penalty of at least 25% on writers who received generative AI ideas, relative stories written only by humans, and most evaluators indicated that the content creators, on which the models were based, should be compensated. Most evaluators also indicated that disclosure of the use of AI or the underlying text from AI should be part of publications that used such tools. Overall, however, most evaluators found the use of AI in writing stories to be ethical and still a “creative act.” These results indicate support for the use of generative AI in creative outputs, with important potential limits on ownership or credit and requirements for disclosure.

Our choice of the experimental design offers a fairly stringent test to measure the causal impact of generative AI on creativity (*34*). We designed our study such that endogenous decisions by the writer are minimized, but not fully eliminated. We do not allow writers to customize the call to the generative AI engine, nor do we allow for repeated interactions between writers and generative AI, both of which may increase the effectiveness and magnitude of the impact of generative AI on creativity. If that is the case, our estimates are likely a lower bound of the potential that generative AI could offer to writers when they are given full control over the AI engine, or when real-time interactions are enabled that help writers with ideation and enhancement further. That a tightly controlled prompt requesting a generative AI idea shows sizable effects on creativity in our study provides a promising starting point for future researchers to delve deeper into customization and personalization of generative AI for different writers (*8*).

We do, however, allow writers to opt into receiving generative AI ideas, rather than assign generative AI ideas to everyone in the generative AI conditions. We do this to ensure that writers are invested in, and receptive to, what generative AI produces. Furthermore, we anticipated that—if offered—the vast majority of participants would take advantage of the option to at least see the generative AI idea, thus minimizing the risk of self-selection affecting our causal estimates. The empirical evidence shows that nearly 9 of 10 people in the generative AI conditions choose to receive at least one generative AI when offered, bolstering our confidence that our results—based on our conservative intention-to-treat analysis that studies the effect of condition regardless of whether writers did or did not choose to request generative AI ideas—allow for a causal interpretation.

Regardless, our study has limitations in that the creative task is constrained in its length (i.e., eight sentences), medium (i.e., writing), and type of output (i.e., short story), and there is no interactiveness with the LLM or variation in prompts. These constraints limit the generalizability and conclusions we can draw from this study. Constraining the task in such a way may constrain the extent to which participants are able to express their creativity and may not generalize to other less-constrained creativity tasks. It is possible that the effect of generative AI ideas would be attenuated for longer stories if the content of generative AI ideas does not sufficiently guide writers. Furthermore, generative AI ideas in different media, such as images or music, may be incorporated in different ways resulting in a different effect. For example, if the exercise related to drawing a picture, perhaps generative AI ideas would not be as effective for individuals with little experience with drawing (as opposed to writing where most people have experience with the task). To this end, we note that the “usefulness” construct in our creativity measure was adapted to fit our context, but future work should revisit both our own definition of usefulness and ensure that it can be adopted across different domains of creativity to best capture this aspect of creativity. At the same time, we did not study or vary the myriad of motivating factors that encourage creativity in the real world. Introducing financial incentives (*10*), encouraging creative problem solutions (*9*, *11*), or simply encouraging creativity for one’s own pleasure may affect the use and integration of generative AI ideas differently.

Fascinating opportunities exist to expand and further develop this research agenda. We believe that a particularly promising experiment would expand the scope of our current study and build on the current and emerging capabilities of generative AI. Future studies might ask participants to write longer literary stories or produce written output in different contexts. For instance, participants may be asked to solve a specific problem through engagement with generative AI, such as coming up with novel and practical product ideas for a specific market or target audience. A future study could also systematically vary the prompts provided to the LLM, including one experimental condition that allows for more open-ended interaction between the participant and the LLM. Last, with our results showing that generative AI professionalizes the writing but reduces the variance in creative outputs, a future study may introduce financial or ranking incentives for specific outcomes, such as being completely novel.

One final area for further exploration pertains to the motivations of the writers to seek out and use LLMs to improve the creativity of their output. In our study, we randomly assigned writers to one of the generative AI conditions to mitigate selection bias. However, the self-selection itself is worth considering in the future. A study that looks at the extent to which writers self-select into using generative AI to improve an earlier draft of a story would demonstrate whether writers choose this form of iterating through their work given perceptions of the value of generative AI and degree of accuracy of self-assessment. However, we caution that self-selection may not be individually optimal or efficient: We asked participants in our study to self-assess the creativity of their stories, but find that they generally do not self-assess accurately. Furthermore, we do not find any correlation between participants who self-assess their stories to be less creative and their use of generative AI, suggesting that participants who would benefit from the technology the most are not more likely to use it.

Much has been written about the potential replacement of human labor by AI (e.g., automation) (*35*–*37*) or a “horse race” between human and AI-generated ideas (*38*–*40*). We focus on the potential complementarities of AI on human creative production. We do so among a sample of relatively “typical” study participants often used in academic studies (which comes with limitations on population representativeness) (*41*)—that is, we do not study professional writers or unusually creative individuals. These individuals remain an important but understudied population segment, for which the effects of generative AI could be transformative in other ways, potentially offering efficiency gains or improved speed of execution (*6*). That said, our results suggest that generative AI may have the largest impact on individuals who are less creative.

While these results point to an increase in individual creativity, there is risk of losing collective novelty. In general equilibrium, an interesting question is whether the stories enhanced and inspired by AI will be able to create sufficient variation in the outputs they lead to. Specifically, if the publishing (and self-publishing) industry were to embrace more generative AI-inspired stories, our findings suggest that the produced stories would become less unique in aggregate and more similar to each other. This downward spiral shows parallels to an emerging social dilemma (*42*): If individual writers find out that their generative AI-inspired writing is evaluated as more creative, they have an incentive to use generative AI more in the future, but by doing so, the collective novelty of stories may be reduced further. In short, our results suggest that despite the enhancement effect that generative AI had on individual creativity, there may be a cautionary note if generative AI were adopted more widely for creative tasks.

Generative AI is a rapidly evolving technology with its full potential yet to be explored. While our study used the most recent version of a widely used LLM—OpenAI’s GPT-4—current technologies and approaches may soon become obsolete. However, rather than limiting our study or future studies, we believe the fast progress of generative AI development and the broad array of questions surrounding the relationship between generative AI and human potential offers exciting opportunities for researchers interested in creativity, innovation, and the arts. If generative AI leads to enhancements of human creativity in a conservatively designed experimental study today, the creative possibilities for tomorrow may extend beyond our current, collective imagination.

## MATERIALS AND METHODS

### Writer study and experimental conditions

For the writer study, we recruited 500 participants to participate in the experiment from the Prolific platform. Using the platform’s filtering options, we included participants who were Prolific participants who indicated that they are based in the United Kingdom with an approval rating of at least 95% from between 100 and 1,000,000 prior submissions. Writers were not selected based on prior writing skills or their creativity. Of the 500 participants who began the study, 169 exited the study before giving consent, 22 were dropped for not giving consent, and 13 dropped out before completing the study. Three participants in the Human-only condition admitted to using generative AI during their story writing exercise and—as per our preregistration—were therefore dropped from the analysis, resulting in a total number of writers and stories of 293.

We first asked each participant to complete the DAT (*25*), a trait measure of creativity. Each participant was then provided with instructions to complete a story writing task. Participants were randomized into writing about one of the following three topics: an adventure on the open seas, an adventure in the jungle, and an adventure on a different planet. Participants (using the “open seas” writing topic as an example) received the following instructions: “We would like you to write a story about an adventure on the open seas. You can write about anything you like. The story must be exactly eight sentences long and it needs to be written in English and appropriate for a teenage and young adult audience (approximately 15 to 24 years of age).”

Participants were randomized into one of three experimental conditions: Human-only, Human with one GenAI idea, and Human with five GenAI ideas. In the Human-only condition, the participant was provided with a text box in which she could provide her response. Automatic checks were conducted to ensure the story meets the length requirements of eight sentences before the participant could continue. In the Human with one GenAI idea condition and the Human with five GenAI ideas conditions, the participant had the option to receive a three-sentence idea for a story from an LLM. When a participant clicked on “Generate Story Idea…,” we passed the following prompt to OpenAI’s GPT API (again, using the open seas topic as an example): “Write a three-sentence summary of a story about an adventure on the open seas.” The response from the API was passed to the participant. At the time of the study, we used the API from OpenAI’s latest model, GPT-4. Those in the Human with one GenAI idea condition could only receive one story idea, while those in the Human with five GenAI ideas condition could receive up to five story ideas, each of which was visible to the participant. Participants were not able to copy and paste the generative AI idea text.

We then asked the writers to evaluate the creativity of their own stories. We asked them how much they agreed with six stylistic statements, including whether they enjoyed writing it, how well written it was, how boring it was, how funny it was, to what extent there was a surprise twist, and whether it changed their expectations of future stories (questions were asked in a random order across participants). We then asked participants about their view of story profits they should receive (as a percentage) and whether the story reflected their own ideas, as well as the novelty and usefulness of the story (on a nine-point scale). We also asked the Human-only condition whether they used AI to help them complete the task. (As described above, if writers in the Human-only condition answer “yes” to this question, they were not included in our main analysis, as per our preregistration. In section S3, we present evidence that suggests that the writers in the Human-only condition likely did not use generative AI outside of the experimental interface.)

Section S6 provides an illustrative overview of the kinds of stories produced by the writers in the three conditions: To provide breadth, we include stories that score at the top, median, and lower ends of the distribution for the novelty and usefulness indices in each condition. Section S7 shows screenshots of the interface presented to writer participants in each of the three conditions.

### Evaluator study

For the evaluator study, the 293 total stories were then evaluated by a separate set of evaluators on Prolific. Using the platform’s filtering options, we included participants who were Prolific participants who indicated that they are based in the United Kingdom with an approval rating of at least 95% from between 100 and 1,000,000 prior submissions and had not previously participated in the writer study. Participants were not selected on the basis of prior experience in the publishing industry, but represent “regular” readers. Each evaluator was shown six stories (two stories from each topic). The evaluations associated with the writers who did not complete the writer study and those in the Human-only condition who acknowledged using AI to complete the story were dropped.

The order in which the stories were presented for review was randomized across evaluators. Evaluators were presented with one story at a time and asked to provide their feedback on the stylistic characteristics, novelty, and usefulness of the story. We presented the evaluator the same stories a second time and asked for an assessment of whether the story was written by a human or AI (as a percentage). We then disclosed whether the writer was offered the opportunity to generate an AI idea and, if so, whether the writer made use of it. If the author did use AI, we provide the evaluator with the text of the idea. Following that disclosure, we asked about the extent to which the story reflects the author’s ideas and the extent to which the author has an ownership claim over the story. If the author used AI, we also asked the share of the profit the author should receive. After all story evaluations, we asked participants to assess six statements about the use of AI in writing stories. Screenshots of the interface presented to evaluator participants are shown in section S8.

There were a total of 3519 evaluations of 293 stories made by 600 evaluators. Four evaluations remained for 5 evaluators, five evaluations remained for 71 evaluators, and all six remained for 524 evaluators. The number of evaluations per story varied because of random assignment of stories to evaluators: One story received 9 reviews, 9 stories received 10 reviews, 61 stories received 11 reviews, 141 stories received 12 reviews, 77 stories received 13 reviews, and 4 stories received 14 reviews.

### Outcome variables

For our preregistered indices, we followed Harvey and Berry’s definition of creativity in terms of novelty and usefulness (*12*), which draws on a diverse range of interpretations of creativity in the literature. Unless otherwise noted, all outcome (dependent) variables were assessed on a nine-point scale from 1 (not at all) to 9 (extremely) to capture disagreement versus agreement with a statement or a question. The exact wording for each statement or question is shown in sections S7 and S8.

#### Creativity

Our novelty index had three components (novel, original, and rare), with which we created an average value. The usefulness index also had three components (appropriate, feasible, and publishable), with which we also created an average value. Cronbach’s α for the novelty and usefulness indices was 0.92 and 0.89, respectively. Furthermore, we explored six additional outcome variables focused on how enjoyable, how well written, how boring, and how funny the story was, as well as whether the story had a surprising twist and whether it had changed what the reader expects of future stories.

#### Characteristics, ownership, and profits

Next, evaluators indicated the extent to which they believed each story was based on inputs from a generative AI tool (on a scale from 0% to 100%). On the following pages, they learned if generative AI was available to writers and then stated the extent to which the writer had ownership over the final story and the extent to which the story reflected the author’s own ideas. These two questions were averaged to create an ownership index. Cronbach’s α for the ownership index was 0.92. In addition, if generative AI was used, evaluators were also asked to choose how to split hypothetical profits between the writer and the creator of the AI tool (on a scale from 0% to 100%).

#### Ethics and use of AI

In the post-experimental survey, evaluators were asked their beliefs and agreement about the ethicality of using AI in producing creative output across six statements. Participants indicated their agreement with statements relating to the extent to which AI use is ethical, whether a story using AI would still count as a "creative act," whether content creators on which the AI idea is based should be compensated, whether AI should be credited, and whether the AI-generated content should be accessible alongside the final story.

#### Similarity scores

We computed measures of the writer’s story to all other stories from writers in the same condition as well as to a generative AI idea. We did so by computing the cosine similarity of the embeddings and multiplying the value by 100 to arrive at a measure that ranges from 0 to 100. Embeddings were obtained via a call to OpenAI’s embeddings API. For generative AI ideas, we first randomly assigned a generative AI story from the same condition among all generative AI ideas to all writers who did not have an idea (i.e., all writers in the Human-only condition and writers in the generative AI idea conditions who opted not to request for any generative AI ideas). For writers who opted to receive multiple generative AI ideas, we selected the first available idea. First, we computed the cosine similarity of the embeddings of the story and the respective generative AI idea. Second, for the similarity measure to all other stories, we took the cosine similarity of the embedding of the focal story with the average embedding for all other stories in the same condition.

### Statistical analysis

Unless otherwise noted, we ran regressions using ordinary least squares (OLS) using robust standard errors for outcomes derived from the writer study (each writer produces one story) and robust standard errors clustered at the participant (i.e., evaluator) level for those derived from the evaluator study (each evaluator assesses six stories). The key independent variables were the conditions to which writers are exogenously assigned where Human-only is the baseline (reference category) and the Human with one GenAI idea and Human with five GenAI ideas conditions are dummy variables.

### Preregistration and ethics approval

The study was preregistered at AsPredicted.org (ID 136723); a copy of the preregistration is included in section S9. The study was approved by the ethics boards at the UCL School of Management (ID UCLSOM-2023-002) and the University of Exeter (ID 1642263). Informed consent was obtained for both the writer study and the evaluator study.

## Supplementary Materials

### This PDF file includes:

Sections S1 to S9 Tables S1 to S18 Figs. S1 to S6
