# Supplementary material for: Generative AI enhances individual creativity but reduces the collective diversity of novel content
Source: Sci Adv. 2024 Jul 12;10(28):eadn5290. doi: 10.1126/sciadv.adn5290 (PMC11244532; doi:10.1126/sciadv.adn5290)
Supplement: Supplementary file 1 — Sections S1 to S9 Tables S1 to S18 Figs. S1 to S6 [file sciadv.adn5290_sm.pdf]

Science Advances
AAAS

Supplementary Materials for

Generative AI enhances individual creativity but reduces the collective diversity of novel content

Anil R. Doshi and Oliver P. Hauser

Corresponding author: Anil R. Doshi, anil.doshi@ucl.ac.uk; Oliver P. Hauser, o.hauser@exeter.ac.uk

Sci. Adv. **10**, eadn5290 (2024)
DOI: 10.1126/sciadv.adn5290

This PDF file includes:

Sections S1 to S9
Tables S1 to S18
Figs. S1 to S6

Section 1. Summary of questions for Writer and Evaluator studies

Participants are asked to indicate to what extent they agree with each statement or question on scale from 1 (not at all) to 9 (extremely).

	**Question text** [blue = evaluator, red = writer]	**Part of an index?**	**Asked of writers?**	**Asked of evaluators?**
1	How novel do you think the / your story is?	Novelty index	✓	✓
2	How original do you think the / your story is?	Novelty index	✓	✓
3	How rare (i.e., unusual) do you think the / your story is?	Novelty index	✓	✓
4	How appropriate do you think the / your story is for the intended audience?	Usefulness index	✓	✓
5	How feasible do you think the / your story is to be developed into a complete book?	Usefulness index	✓	✓
6	How likely do you think it would be that the / your story is turned into a complete book if a publisher read it and hired a professional author to expand on the idea?	Usefulness index	✓	✓
7	I enjoyed reading / writing this story.		✓	✓
8	This story is well written.		✓	✓
9	This story is boring.		✓	✓
10	This story has changed what I expect of future stories I will read.		✓	✓
11	This story is funny.		✓	✓

	**Question text** [blue = evaluator, red = writer]	**Part of an index?**	**Asked of writers?**	**Asked of evaluators?**
12	This story has a surprising twist.		✓	✓
13	Please indicate to what extent this specific AI generated idea affected the story you submitted.		✓	
14	Please indicate the extent (if any) to which you think this story was based on inputs from an AI tool (e.g. ChatGPT or similar generative AI tool). (0% to 100% scale)			✓
15	To what extent do you think the / your story reflects the author's / your own ideas?	Ownership index (Evaluators)	✓	✓
16	To what extent does the author have an "ownership" claim to the final story?	Ownership index (Evaluators)		✓
17	If this story were published and sold tomorrow, how much of the story's profit do you believe should belong to (you / the author) versus the creators of the generative AI tool that may have provided the starting point for the story? (0% to 100% scale)			✓
18	Relying on the use of AI to write a new story is unethical.			✓
19	If AI is used in any part of the writing of a story, the final story no longer counts as a "creative act".			✓
20	It is ethically acceptable to use AI to come up with an initial idea for a story.			✓
21	It is ethically acceptable to use AI to write an entire story without acknowledging the use of AI.			✓

	**Question text** [blue = evaluator, red = writer]	**Part of an index?**	**Asked of writers?**	**Asked of evaluators?**
22	If AI is used in any part of the writing of a story, the creators of the content on which the AI output was based on should be compensated.			✓
23	If a human creator (author) uses AI in part of the writing of a story, the AI-generated content should be accessible alongside the final story.			✓

Section 2. Supporting Tables and Figures

Table S1. Comparison of Means for writers

	Human	1AI idea	5AI ideas	Human / 1AI idea	Human / 5AI ideas	1AI idea / 5AI ideas
	mean	mean	mean	p	p	p
writer DAT score	77.617	76.868	77.254	(0.415)	(0.698)	(0.683)
writer creative	5.505	5.740	5.673	(0.473)	(0.601)	(0.832)
writer creative job	4.747	4.160	4.612	(0.109)	(0.707)	(0.203)
writer tech comfort	7.147	6.870	6.878	(0.290)	(0.252)	(0.978)
writer AI engagement	4.232	4.870	4.796	(0.061)	(0.093)	(0.823)
writer used ChatGPT	0.484	0.630	0.561	(0.041)	(0.287)	(0.327)
writer used text AI tools	0.474	0.590	0.571	(0.105)	(0.176)	(0.792)
writer used image AI tools	0.274	0.300	0.265	(0.686)	(0.896)	(0.590)
writer used audio AI tools	0.053	0.110	0.061	(0.143)	(0.798)	(0.222)
writer used music AI tools	0.053	0.060	0.041	(0.824)	(0.699)	(0.539)
writer used video AI tools	0.042	0.020	0.020	(0.379)	(0.391)	(0.984)
writer gender female	0.379	0.440	0.408	(0.389)	(0.680)	(0.652)
writer income > £50,000	0.158	0.080	0.133	(0.095)	(0.621)	(0.232)
writer education undergrad +	0.168	0.170	0.204	(0.977)	(0.527)	(0.541)
writer employed part- or full-time	0.779	0.750	0.724	(0.636)	(0.384)	(0.685)
writer age	38.526	41.050	39.041	(0.170)	(0.785)	(0.282)
Observations	95	100	98	195	193	198

Table S2. Comparison of means for evaluators (selecting on condition of first story evaluated)

	Human	1AI idea	5AI ideas	Human / 1AI idea	Human / 5AI ideas	1AI idea / 5AI ideas
	mean	mean	mean	p	p	p
evaluator creative	5.648	5.522	5.580	(0.562)	(0.749)	(0.793)
evaluator creative job	4.528	4.507	4.395	(0.936)	(0.599)	(0.654)
evaluator tech comfort	7.171	7.030	6.935	(0.370)	(0.135)	(0.556)
evaluator AI engagement	4.598	4.368	4.420	(0.351)	(0.468)	(0.830)
evaluator used ChatGPT	0.633	0.602	0.600	(0.522)	(0.497)	(0.968)
evaluator used text AI tools	0.643	0.562	0.625	(0.098)	(0.707)	(0.201)
evaluator used image AI tools	0.347	0.333	0.285	(0.778)	(0.186)	(0.296)
evaluator used audio AI tools	0.075	0.065	0.070	(0.676)	(0.837)	(0.832)
evaluator used music AI tools	0.055	0.050	0.075	(0.805)	(0.426)	(0.297)
evaluator used video AI tools	0.075	0.040	0.045	(0.128)	(0.203)	(0.797)
evaluator gender female	0.467	0.468	0.480	(0.995)	(0.801)	(0.805)
evaluator income > £50,000	0.141	0.174	0.165	(0.360)	(0.501)	(0.808)
evaluator education undergrad +	0.211	0.214	0.170	(0.944)	(0.298)	(0.265)
evaluator employed part-/full-time	0.724	0.811	0.750	(0.039)	(0.551)	(0.141)
evaluator age	40.206	39.697	39.065	(0.692)	(0.380)	(0.623)

Observations	199	201	200	400	399	401

Table S3. Evaluator assessment of creativity (separate AI idea conditions)

	(1)	(2)	(3)	(4)	(5)	(6)	(7)	(8)
	Novelty index	Novel	Original	Rare	Usefulness index	Appropriate	Feasible	Publishable
Human with 1 GenAI idea	0.207^{*}	0.215^{*}	0.168^{+}	0.237^{*}	0.185^{*}	0.132	0.193^{+}	0.230^{*}
	(0.089)	(0.096)	(0.094)	(0.093)	(0.090)	(0.090)	(0.101)	(0.105)
Human with 5 GenAI ideas	0.311^{***}	0.304^{**}	0.339^{***}	0.289^{**}	0.453^{***}	0.324^{***}	0.518^{***}	0.518^{***}
	(0.087)	(0.095)	(0.093)	(0.089)	(0.090)	(0.092)	(0.100)	(0.106)
Constant	3.851^{***}	4.023^{***}	3.972^{***}	3.559^{***}	5.023^{***}	5.708^{***}	4.810^{***}	4.551^{***}
	(0.076)	(0.083)	(0.081)	(0.078)	(0.073)	(0.078)	(0.082)	(0.086)
Observations	3519	3519	3519	3519	3519	3519	3519	3519
F-Stat	6.42	5.18	6.65	5.68	13.2	6.35	14.1	12.3
Adj R-squared	0.0033	0.0028	0.0032	0.0029	0.0073	0.0032	0.0075	0.0070

Note: + $p < 0.10$, * $p < 0.05$, ** $p < 0.01$, *** $p < 0.001$.

Table S4. Evaluator assessment of creativity (robustness checks)

	(1)	(2)	(3)	(4)	(5)	(6)	(7)	(8)
	Novelty index	Novelty index	Novelty index	Novelty index	Usefulness index	Usefulness index	Usefulness index	Usefulness index
Human with 1 GenAI idea	0.204^{**}	0.203^{**}	0.131^{+}	0.246^{+}	0.246^{**}	0.248^{**}	0.215^{*}	0.243^{+}
	(0.079)	(0.078)	(0.077)	(0.134)	(0.084)	(0.083)	(0.083)	(0.135)
Human with 5 GenAI ideas	0.355^{***}	0.354^{***}	0.322^{***}	0.455^{**}	0.538^{***}	0.540^{***}	0.536^{***}	0.569^{***}
	(0.078)	(0.077)	(0.076)	(0.146)	(0.084)	(0.084)	(0.084)	(0.149)
Used AI				-0.142				-0.035
				(0.128)				(0.129)
Constant	3.837^{***}	3.590^{***}	3.561^{***}	3.561^{***}	4.974^{***}	5.145^{***}	5.198^{***}	5.198^{***}
	(0.047)	(0.076)	(0.084)	(0.084)	(0.050)	(0.083)	(0.095)	(0.095)
Story order fixed effects	No	Yes	Yes	Yes	No	Yes	Yes	Yes
Story topic fixed effects	No	No	Yes	Yes	No	No	Yes	Yes
Evaluator fixed effects	Yes	Yes	Yes	Yes	Yes	Yes	Yes	Yes
Observations	3519	3519	3519	3519	3519	3519	3519	3519
F-Stat	10.4	6.08	11.5	10.5	21.3	7.02	6.43	5.79
Adj R-squared	0.0068	0.012	0.033	0.033	0.014	0.016	0.019	0.019

Note: + $p < 0.10$, * $p < 0.05$, ** $p < 0.01$, *** $p < 0.001$.

Table S5. Writer self-evaluation of creativity

	(1)	(2)	(3)	(4)	(5)	(6)	(7)	(8)
	writer novel index	story novel	story original	story rare	writer useful index	story appropriate	story feasible	story publishable
Human with 1 GenAI idea	-0.025	-0.077	-0.053	0.054	0.078	0.237	-0.271	0.267
	(0.292)	(0.318)	(0.335)	(0.317)	(0.251)	(0.200)	(0.352)	(0.365)
Human with 5 GenAI ideas	-0.206	-0.196	-0.455	0.033	0.170	-0.018	0.189	0.339
	(0.295)	(0.316)	(0.331)	(0.311)	(0.251)	(0.201)	(0.351)	(0.370)
Constant	4.505***	4.737***	4.863***	3.916***	5.779***	7.263***	5.821***	4.253***
	(0.214)	(0.236)	(0.244)	(0.223)	(0.176)	(0.135)	(0.249)	(0.263)
Observations	293	293	293	293	293	293	293	293
F-Stat	0.30	0.20	1.18	0.015	0.23	0.94	0.87	0.47
Adj R-squared	-0.0048	-0.0055	0.0010	-0.0068	-0.0053	-0.00012	-0.00082	-0.0036

Note: + $p < 0.10$, * $p < 0.05$, ** $p < 0.01$, *** $p < 0.001$.

Table S6. Evaluator assessment of emotional characteristics

	(1)	(2)	(3)	(4)	(5)	(6)
	Well written	Enjoyed	Funny	Future	Twist	Boring
Human with 1 GenAI idea	0.120	0.216*	-0.059	0.138	0.384***	-0.060
	(0.096)	(0.098)	(0.069)	(0.085)	(0.103)	(0.097)
Human with 5 GenAI ideas	0.372***	0.375***	-0.106	0.251**	0.468***	-0.200*
	(0.098)	(0.097)	(0.067)	(0.089)	(0.100)	(0.102)
Constant	4.677***	4.512***	2.085***	3.042***	3.414***	4.258***
	(0.081)	(0.080)	(0.060)	(0.083)	(0.083)	(0.081)
Observations	3519	3519	3519	3519	3519	3519
F-Stat	7.87	7.48	1.25	3.97	11.8	2.07
Adj R-squared	0.0040	0.0040	0.00019	0.0018	0.0065	0.00068

Note: + $p < 0.10$, * $p < 0.05$, ** $p < 0.01$, *** $p < 0.001$.

Table S7. Writer self-evaluation of emotional characteristics

	(1)	(2)	(3)	(4)	(5)	(6)
	writer well written	writer enjoyed	writer funny	writer future	writer twist	writer boring
Human with 1 GenAI idea	0.059	-0.171	-0.425	-0.274	0.177	0.022
	(0.281)	(0.257)	(0.289)	(0.286)	(0.376)	(0.300)
Human with 5 GenAI ideas	-0.146	-0.153	-0.401	-0.161	0.272	0.276
	(0.280)	(0.232)	(0.282)	(0.299)	(0.365)	(0.288)
Constant	5.421***	7.011***	3.105***	3.274***	4.453***	3.968***
	(0.197)	(0.183)	(0.210)	(0.206)	(0.268)	(0.208)
Observations	293	293	293	293	293	293
F-Stat	0.28	0.28	1.36	0.46	0.28	0.57
Adj R-squared	-0.0049	-0.0048	0.0029	-0.0039	-0.0050	-0.0031

Note: + $p < 0.10$, * $p < 0.05$, ** $p < 0.01$, *** $p < 0.001$.

Table S8. Writer creativity and accessing generative AI Ideas

	(1)	(2)
	Used AI	Used AI
Model:	OLS	Logistic
Human with 5 GenAI ideas	0.306	8.938
	(0.483)	(8.178)
writer DAT score	-0.003	-0.021
	(0.005)	(0.034)
Human with 5 GenAI ideas # writer DAT score	-0.002	-0.094
	(0.006)	(0.100)
Constant	1.048**	3.134
	(0.349)	(2.617)
Observations	197	197
F-Stat / Wald Chi-squared (logistic)	4.00	6.58
Adj R-squared / Pseudo R-squared (logistic)	0.034	0.081

Note: + $p < 0.10$, * $p < 0.05$, ** $p < 0.01$, *** $p < 0.001$.

Table S9. Evaluator assessment of creativity (conditions interacted with writer's DAT score)

	(1)	(2)	(3)	(4)	(5)	(6)	(7)	(8)
	Novelty index	Novel	Original	Rare	Usefulness index	Appropriate	Feasible	Publishable
Human with 1 GenAI idea	0.273	0.673	-0.766	0.911	2.633*	2.643*	2.457*	2.798*
	(1.014)	(1.095)	(1.081)	(1.027)	(1.090)	(1.153)	(1.184)	(1.250)
Human with 5 GenAI ideas	2.528**	2.621*	2.378*	2.586*	3.966***	3.721***	3.844***	4.331***
	(0.969)	(1.039)	(1.045)	(1.010)	(1.024)	(1.082)	(1.138)	(1.197)
writer DAT score	0.038***	0.040***	0.035***	0.040***	0.045***	0.044***	0.044***	0.048***
	(0.010)	(0.010)	(0.010)	(0.010)	(0.010)	(0.011)	(0.012)	(0.012)
Human with 1 GenAI idea # writer DAT score	-0.001	-0.006	0.012	-0.008	-0.032*	-0.032*	-0.029^{+}	-0.033*
	(0.013)	(0.014)	(0.014)	(0.013)	(0.014)	(0.015)	(0.015)	(0.016)
Human with 5 GenAI ideas # writer DAT score	-0.028*	-0.030*	-0.026^{+}	-0.029*	-0.045***	-0.044**	-0.043**	-0.049**
	(0.012)	(0.013)	(0.013)	(0.013)	(0.013)	(0.014)	(0.015)	(0.015)
Constant	0.857	0.898	1.244	0.428	1.511^{+}	2.344**	1.380	0.808
	(0.753)	(0.815)	(0.813)	(0.764)	(0.806)	(0.857)	(0.907)	(0.915)
Observations	3494	3494	3494	3494	3494	3494	3494	3494
F-Stat	9.18	7.46	9.43	8.58	8.72	5.26	8.41	8.20
Adj R-squared	0.012	0.010	0.012	0.011	0.013	0.0076	0.012	0.012

Note: + $p < 0.10$, * $p < 0.05$, ** $p < 0.01$, *** $p < 0.001$.

Table S10. Evaluator assessment of emotional characteristics (conditions interacted with DAT score)

	(1)	(2)	(3)	(4)	(5)	(6)
	Well written	Enjoyed	Funny	Future	Twist	Boring
Human with 1 GenAI idea	1.477	1.017	-1.804*	0.983	1.760	-2.147+
	(1.166)	(1.119)	(0.739)	(1.092)	(1.211)	(1.216)
Human with 5 GenAI ideas	4.717***	3.629***	0.488	1.915+	2.461*	-3.531**
	(1.075)	(1.083)	(0.766)	(1.042)	(1.105)	(1.128)
writer DAT score	0.052***	0.042***	0.003	0.032**	0.057***	-0.051***
	(0.011)	(0.011)	(0.007)	(0.010)	(0.011)	(0.011)
Human with 1 GenAI idea # writer DAT score	-0.017	-0.010	0.023*	-0.011	-0.018	0.027+
	(0.015)	(0.014)	(0.010)	(0.014)	(0.016)	(0.016)
Human with 5 GenAI ideas # writer DAT score	-0.056***	-0.042**	-0.007	-0.021	-0.026+	0.043**
	(0.014)	(0.014)	(0.010)	(0.013)	(0.014)	(0.014)
Constant	0.644	1.255	1.846**	0.582	-1.005	8.253***
	(0.828)	(0.828)	(0.586)	(0.802)	(0.843)	(0.876)
Observations	3494	3494	3494	3494	3494	3494
F-Stat	9.95	7.68	4.34	4.80	15.8	5.80
Adj R-squared	0.013	0.010	0.0029	0.0060	0.019	0.0076

Note: + $p < 0.10$, * $p < 0.05$, ** $p < 0.01$, *** $p < 0.001$.

Table S11. Writer story similarity

	(1)	(2)
	story similarity to other stories in condition	story AI Idea similarity (incl simulated ideas)
Human with 1 GenAI idea	0.871***	4.288***
	(0.227)	(0.614)
Human with 5 GenAI ideas	0.718**	4.105***
	(0.240)	(0.577)
Constant	89.961***	82.850***
	(0.161)	(0.343)
Observations	293	293
F-Stat	8.23	37.3
Adj R-squared	0.044	0.16

Note: + $p < 0.10$, * $p < 0.05$, ** $p < 0.01$, *** $p < 0.001$.

Figure S1. Violin plot of conditions

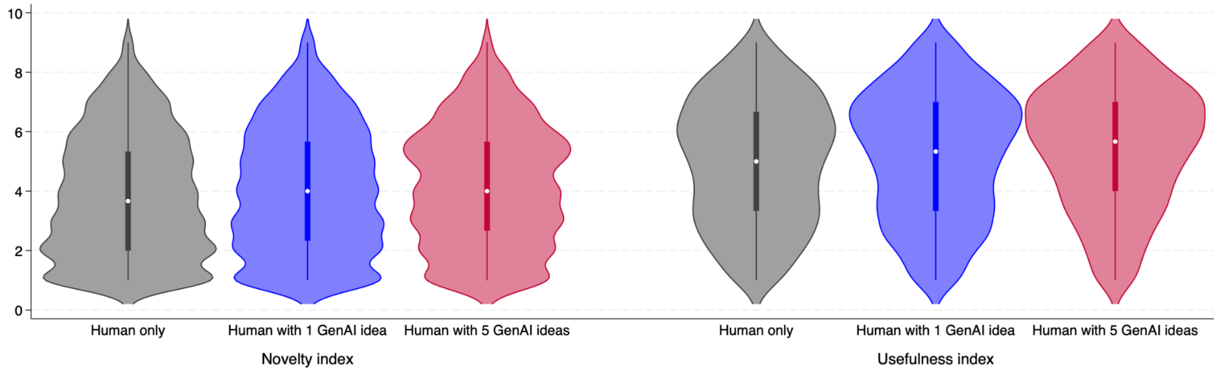

Figure S2. Remainder of emotion outcomes by inherent creativity

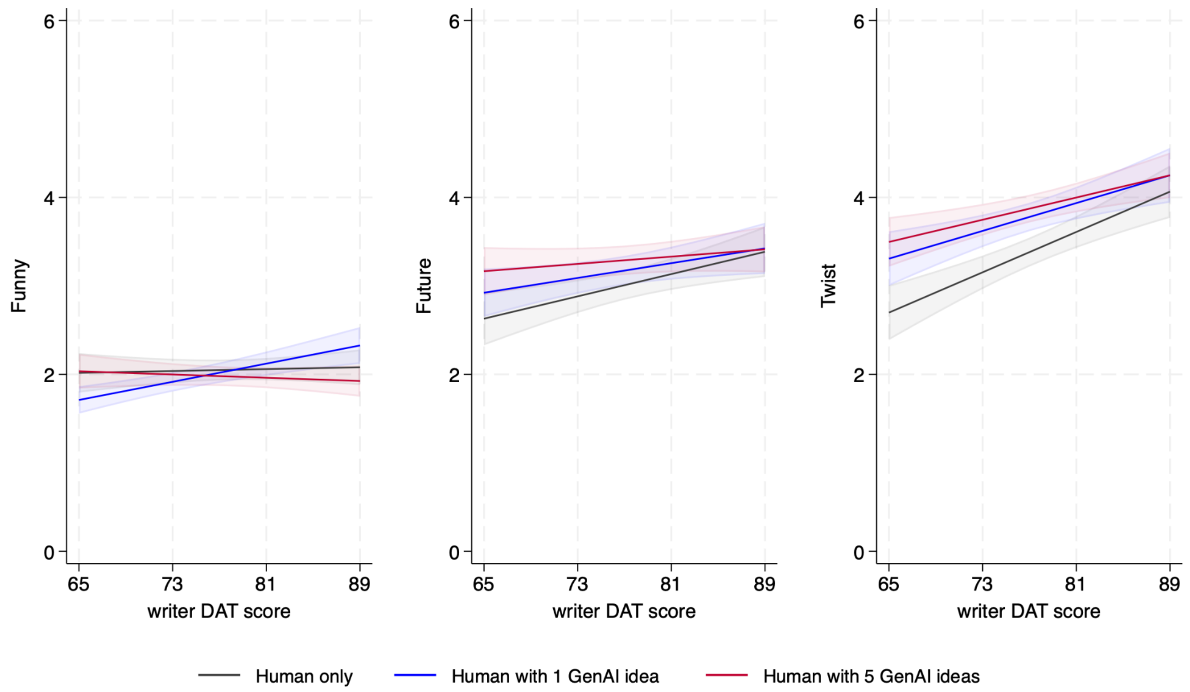

Section 3. Similarity to AI ideas of *Human only* participants and generative AI participants who did not use generative AI

One concern is that participants in the *Human only* condition reported that they did not use AI, but did in fact do so. We conduct the following analysis, which suggests this is not the case. For the *Human only* condition and those in the generative AI idea conditions that did not opt to use generative AI, we provide a “simulated” AI story idea. We do this by randomly selecting one of the generative AI ideas that were generated for that topic and allocating it to participants who could not or did not access a generative AI idea. Then we look at the distribution of cosine similarity between a participant’s story and their generative AI story idea. We compare three groups: participants in the *Human only* condition who were randomly assigned a simulated generative AI idea for this analysis, participants in the generative AI idea conditions who did not use generative AI who were randomly assigned a simulated generative AI idea for this analysis, and participants in the generative AI idea conditions who used the generative AI idea during the study, comparing their final story to the first available generative AI idea.

A comparison of the distributions (Figure S4) shows that the mode and range of the first two groups are more similar—stories of *Human only* participants look more like participants who chose not to generate a generative AI story. Those two distributions are less similar to their assigned generative AI ideas than the third group. Summary statistics of the three groups (Table S12) reflect this comparison as well.

Table S12. Summary statistics for Story–AI similarity

	Count	Mean	S.D.	25th pctile	50th pctile	75th pctile
Human	95	82.85	3.34	80.91	82.44	84.83
No AI ideas	23	82.89	3.10	81.45	82.21	84.15
Used AI	175	87.59	4.77	84.24	87.28	90.69

Figure S3. Distribution of Story–AI idea similarity

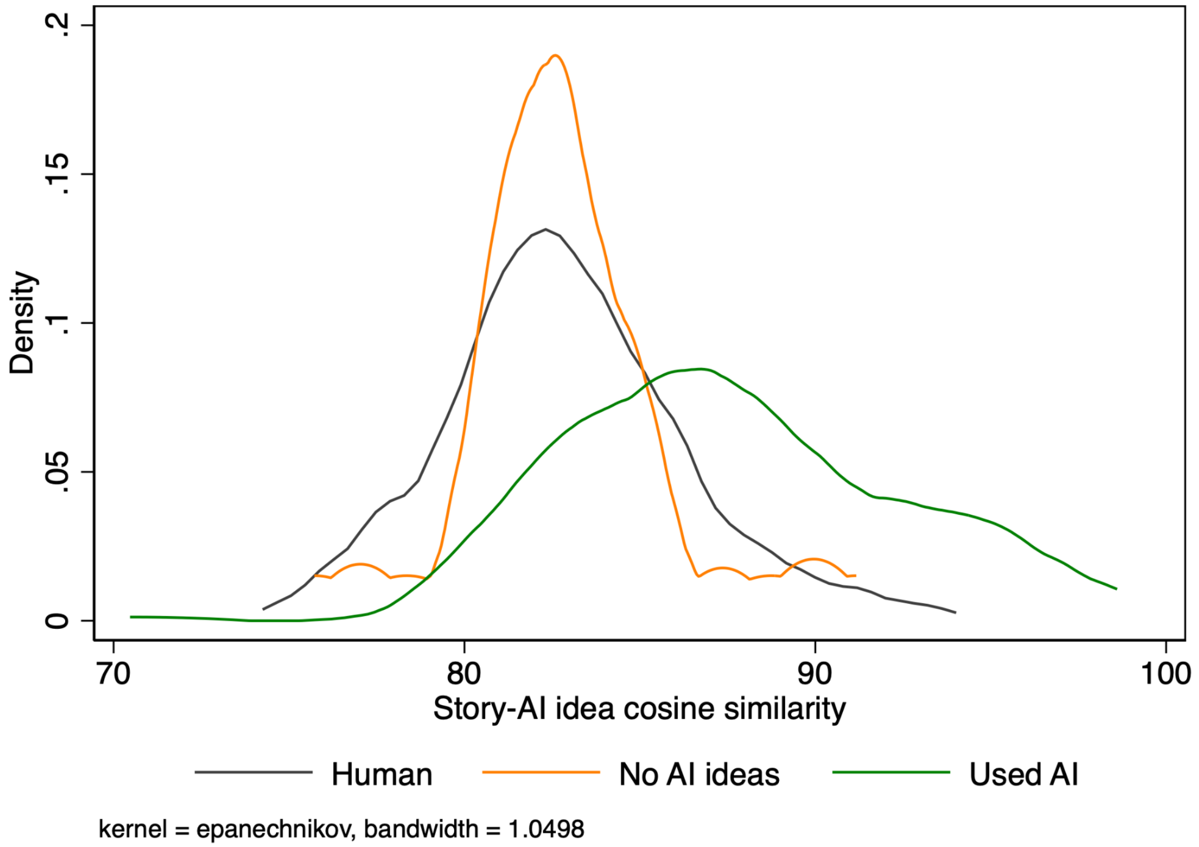

Section 4: Novelty and usefulness of *Human only* versus both generative AI conditions combined

Following our pre-registration, we estimate whether—relative to the Human only baseline condition—the generative AI conditions combined causally affect evaluators' third-party assessments and writers' self-assessments of the stories' creativity, in terms of two commonly used dimensions, novelty and usefulness.

We find that evaluators assess that stories composed by writers in the two generative AI conditions are more creative, in terms of both novelty and usefulness (Figure S4; Figure S5 shows violin plot of raw data). We find that the provision of AI ideas improves the story's novelty by 6.7% ($b=0.259$, $p=0.001$, see Table S13; compared to the Human only mean of 3.85) and its usefulness by 6.4% ($b=0.319$, $p<0.001$; compared to the Human only mean of 5.02). Results for each of the constituent components of the novelty index (whether the story is novel, original, and rare) and usefulness index (whether the story is appropriate, feasible, and publishable) are consistent with the aggregate results for the indices (see Table S13).

Table S13. Evaluator assessment of creativity (combined AI idea conditions)

	(1)	(2)	(3)	(4)	(5)	(6)	(7)	(8)
	Novelty index	Novel	Original	Rare	Usefulness index	Appropriate	Feasible	Publishable
Human with GenAI idea(s)	0.259**	0.259**	0.253**	0.263**	0.319***	0.228**	0.355***	0.374***
	(0.078)	(0.085)	(0.082)	(0.080)	(0.080)	(0.080)	(0.089)	(0.094)
Constant	3.851***	4.023***	3.972***	3.559***	5.023***	5.708***	4.810***	4.551***
	(0.076)	(0.083)	(0.081)	(0.078)	(0.073)	(0.078)	(0.082)	(0.086)
Observations	3519	3519	3519	3519	3519	3519	3519	3519
F-Stat	10.9	9.26	9.47	10.7	16.0	8.04	16.0	16.0
Adj R-squared	0.0032	0.0028	0.0025	0.0031	0.0048	0.0022	0.0047	0.0050

Note: + $p < 0.10$, * $p < 0.05$, ** $p < 0.01$, *** $p < 0.001$.

Figure S4. Comparison of Human only condition to combined generative AI conditions

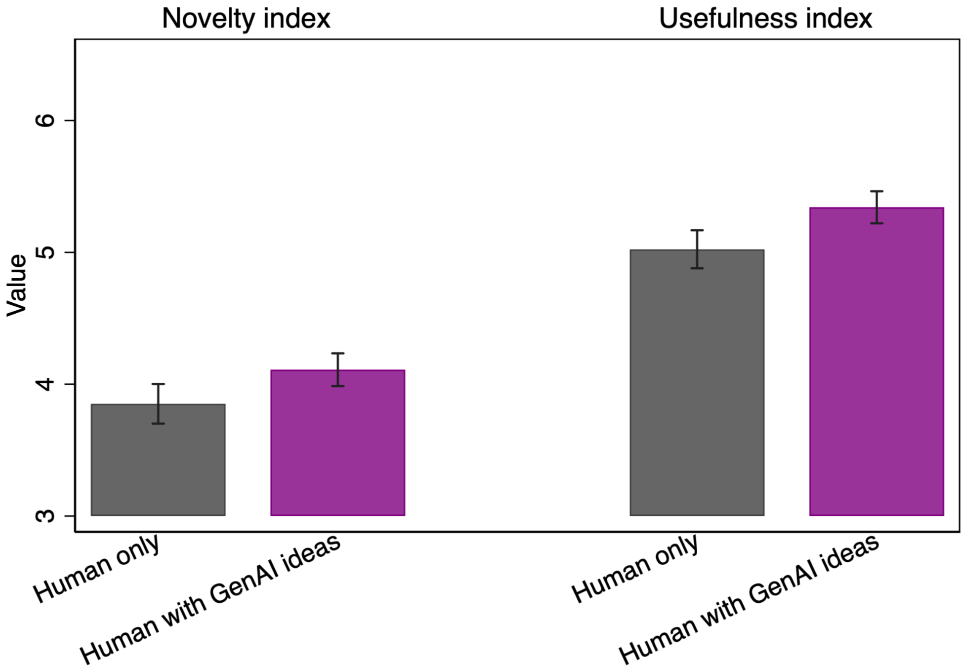

Figure S5. Violin plot of Human only and generative AI conditions (combined)

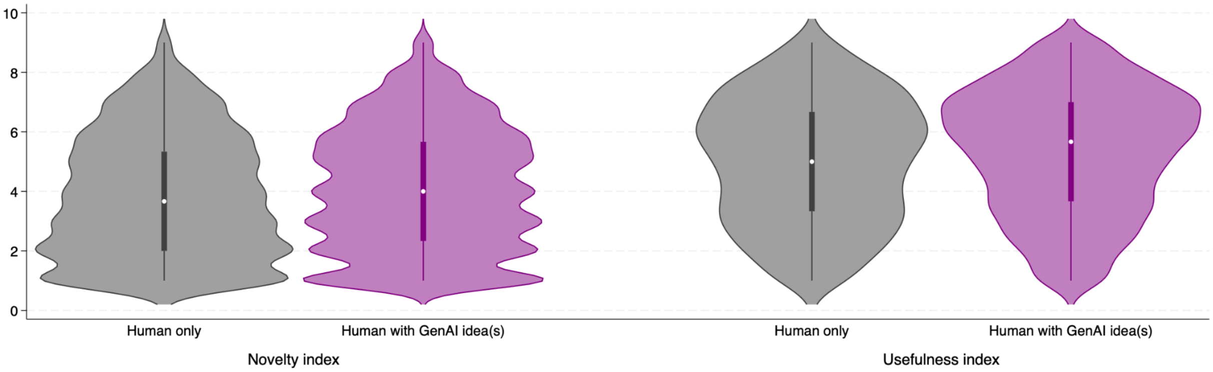

Section 5. Additional findings on ownership attribution and evaluator attitudes towards ethics and generative AI

Identify influence of generative AI. After asking evaluators to review each story for creativity and their emotional reactions, we ask evaluators to estimate to what extent each of the stories they just evaluated may have been influenced by generative AI (on a 100-point scale). We find that evaluators are able to assess whether stories received AI assistance (*Human with 1 GenAI idea*: b=6.21, p<0.001; *Human with 5 GenAI ideas*: b=4.96, p<0.001; see Table S14), though we do not find that they are more or less likely to attribute stories from writers with access to 5 generative AI ideas as being affected by generative AI, compared to those with access to 1 generative AI idea (p=0.3053).

Ownership and profit-sharing. Next, we disclose to evaluators whether generative AI ideas were made available to the writer of each story (*13*) and, if so, the generative AI ideas are shown alongside the final story. We ask evaluators how much ownership should be attributed to the writers for their final story, an index we compute based on their answers to questions about the extent to which a story reflects the writer's own ideas and their claim to ownership (Figure S5). Figure S5A shows that, for stories that were produced by writers who had access to generative AI, evaluators attribute substantially less ownership to the writer. Using the ownership index, evaluators ascribed 25.4% less ownership to authors who had access to one generative AI idea, relative to writers in the *Human only* condition (b = –1.96, p<0.001, see Table S15; compared to the *Human only* mean of 7.74). The ownership discount is even higher for writers who had access to up to five generative AI ideas, at 31.0% (b = –2.40, p<0.001).

Following the questions on ownership, we elicit beliefs from evaluators about how hypothetical profits from selling the short story should be shared. We ask this question only for stories in the *Human with 1 AI idea* and *Human with 5 AI ideas* conditions and only if the writer requested at least one generative AI idea. We ask evaluators to indicate what percent of the story's profits should belong to the writer of the story versus the creator of the generative AI tool. We find that evaluators only impose a marginally significant penalty of 2.3% to writers who had access to 5 generative AI ideas (b= –2.30, p=0.072, see Table S16), relative to having access to 1 generative AI idea. Furthermore, this weak relationship is no longer statistically significant when we include as a control variable the extent to which the evaluator ascribes ownership of the story to the writer in both conditions. A one standard deviation increase in the ownership index results in an additional 16.2% of profits allocated to the writers (b=7.70, p<0.001).

Generative AI and ethics in the creative process. We ask evaluators to indicate to what extent, and how, generative AI should be used to inspire stories in the future. We are interested in understanding the extent to which participants believe using generative AI is ethical and should be credited in the creative process. The responses to six exploratory questions are summarized in Figure S5B.

Evaluators in our sample tended to disagree with the ideas that the use of generative AI in story writing is unethical (52.7% scored 1 to 4 versus 35.7% scored 6 to 9; we focus on choices other than 5, since 5 is the scale midpoint, which might represent a neutral—i.e., indecisive—stance; see Tables S17 and S18) and that the story ceases to be a “creative act” if AI is used in any part of the story writing process (54.5% versus 33.8%).

However, according to evaluators, there were limits in the acceptability of the use of AI. While evaluators tended to agree that using AI for an initial idea was acceptable (26.5% versus 58.7%), they overwhelmingly tended to disagree with the idea that AI could be used for a story without acknowledgement of its use (70.2% versus 20.2%). There was also consensus that the content creators on which the AI output was based ought to be compensated (32.2% versus 51.7%) and, to a lesser extent, that the AI-generated content should be disclosed alongside the final story (35.3% versus 46.7%).

Table S14. Evaluator assessment of AI assistance in story writing

	(1)
	AI assistance
Human with 1 GenAI idea	6.207***
	(1.308)
Human with 5 GenAI ideas	4.955***
	(1.376)
Constant	42.363***
	(1.115)
Observations	3519
F-Stat	11.8
Adj R-squared	0.0067

Note: + $p < 0.10$, * $p < 0.05$, ** $p < 0.01$, *** $p < 0.001$.

Table S15. Evaluator assessment of ownership

	(1)	(2)	(3)
	Ownership index	authors ideas	ownership claim
Human with 1 GenAI idea	-1.962***	-2.021***	-1.902***
	(0.097)	(0.103)	(0.102)
Human with 5 GenAI ideas	-2.401***	-2.462***	-2.341***
	(0.097)	(0.100)	(0.107)
Constant	7.736***	7.628***	7.843***
	(0.075)	(0.077)	(0.078)
Observations	3519	3519	3519
F-Stat	332.4	325.9	266.9
Adj R-squared	0.20	0.20	0.17

Note: + $p < 0.10$, * $p < 0.05$, ** $p < 0.01$, *** $p < 0.001$.

Table S16. Evaluator assessment of profit for writer (versus AI)

	(1)	(2)	(3)	(4)
	profit share	profit share	profit share	profit share
Human with 5 GenAI ideas	-2.300^{+}	-0.913	-0.971	-1.276
	(1.276)	(1.067)	(1.045)	(1.143)
Ownership index		7.702***		
		(0.377)		
ownership claim			7.267***	
			(0.349)	
authors ideas				5.775***
				(0.417)
Constant	61.009***	19.468***	20.548***	30.868***
	(1.233)	(2.115)	(1.957)	(2.509)
Observations	2089	2089	2089	2089
F-Stat	3.25	213.8	219.8	98.8
Adj R-squared	0.0010	0.31	0.34	0.19

Note: + $p < 0.10$, * $p < 0.05$, ** $p < 0.01$, *** $p < 0.001$.

Table S17. Summary statistics of survey responses

	Mean	S.D.	25th pctile	50th pctile	75th pctile
Relying on the use of AI to write a new story is unethical.	4.45	2.54	2.00	4.00	7.00
If AI is used in any part of the writing of a story, the final story no longer counts as a "creative act".	4.37	2.49	2.00	4.00	6.50
It is ethically acceptable to use AI to come up with an initial idea for a story.	5.83	2.36	4.00	6.00	8.00
It is ethically acceptable to use AI to write an entire story without acknowledging the use of AI.	3.27	2.45	1.00	2.00	5.00
If AI is used in any part of the writing of a story, the creators of the content on which the AI output was based on should be compensated.	5.28	2.40	3.00	6.00	7.00
If a human creator (author) uses AI in part of the writing of a story, the AI-generated content should be accessible alongside the final story.	5.08	2.36	3.00	5.00	7.00

Note: $n = 600$.

Table S18. Heatmap of survey response counts (by question)

	Response level								
	1	2	3	4	5	6	7	8	9
Relying on the use of AI to write a new story is unethical.	88	89	81	58	70	53	66	54	41
If AI is used in any part of the writing of a story, the final story no longer counts as a “creative act”.	92	87	76	72	70	53	60	60	30
It is ethically acceptable to use AI to come up with an initial idea for a story.	32	48	36	43	89	68	121	80	83
It is ethically acceptable to use AI to write an entire story without acknowledging the use of AI.	199	116	71	35	58	37	33	22	29
If AI is used in any part of the writing of a story, the creators of the content on which the AI output was based on should be compensated.	58	52	47	36	97	96	108	50	56
If a human creator (author) uses AI in part of the writing of a story, the AI-generated content should be accessible alongside the final story.	57	60	57	38	108	96	90	47	47

Figure S6. Evaluator attitudes toward AI

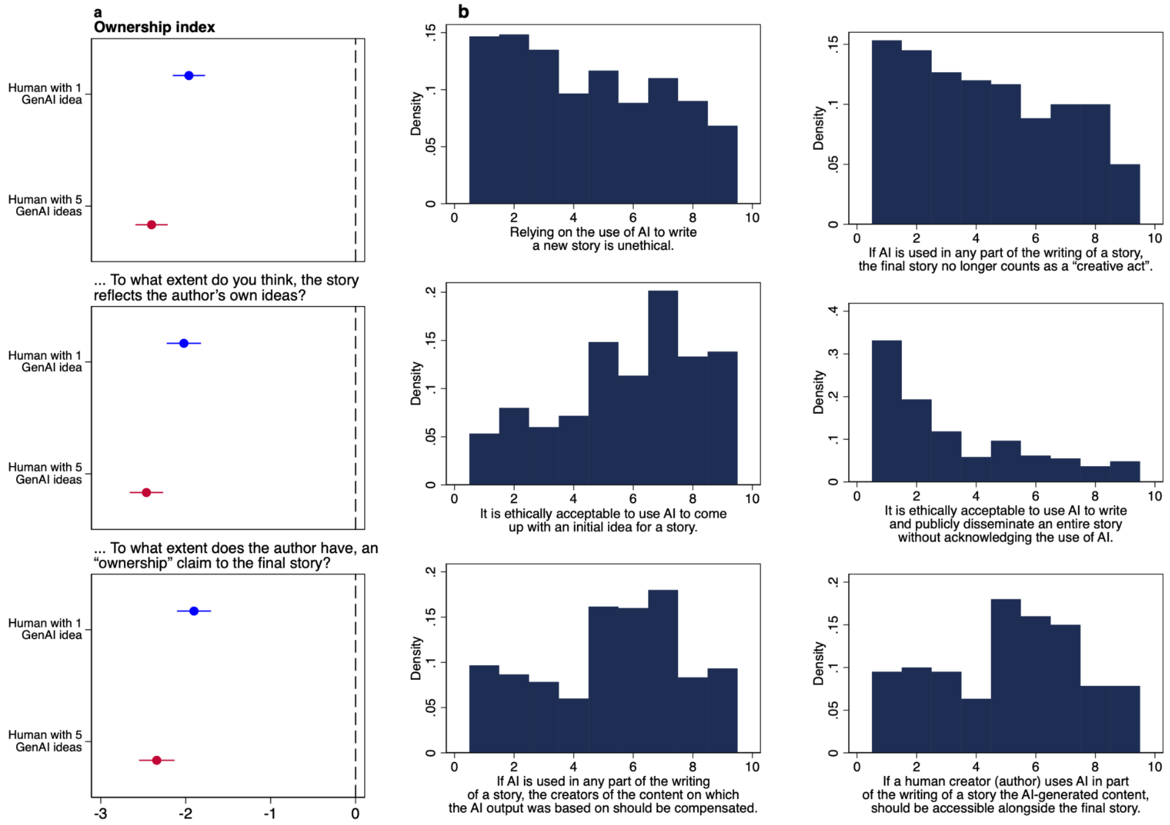

Note. Evaluator assessment of ownership and attitudes toward generative AI. **a**, Compares ownership index (and constituent components) of *Human only* (reference category) to humans with access to 1 and 5 generative AI ideas. **b**, summary of evaluator survey responses on attitudes toward generative AI in creativity output.

Section 6: Illustrative stories of varying novelty and usefulness by condition

In this section, we provide some illustrative examples of the complete stories produced by writers. For both the novelty and usefulness indices, an average across all evaluators was computed. Three stories that were (a) the highest rated, (b) at or near the median, and (c) the lowest rated were included for both measures across each condition. A total of 48 unique stories (out of the 293 stories in the sample) are presented below (six duplicate stories appear for both measures).

The following summarizes the remainder of this section:

	**Human only**	**Human with 1 GenAI idea**	**Human with 5 GenAI ideas**
**Highest**	Novelty: Section 5.1<br>Usefulness: Section 5.10	Novelty: Section 5.4<br>Usefulness: Section 5.13	Novelty: Section 5.7<br>Usefulness: Section 5.16
**Median**	Novelty: Section 5.2<br>Usefulness: Section 5.11	Novelty: Section 5.5<br>Usefulness: Section 5.14	Novelty: Section 5.8<br>Usefulness: Section 5.17
**Lowest**	Novelty: Section 5.3<br>Usefulness: Section 5.12	Novelty: Section 5.6<br>Usefulness: Section 5.15	Novelty: Section 5.9<br>Usefulness: Section 5.18

Section 6.1. Human only, Highest mean novelty

Topic: jungle, Mean novelty index: 5.81

“Catherine turned over in bed and fell and fell and fell for what seemed like forever until, with a thump, she landed on a bed of wet leaves. It was her favourite dream, the jungle dream: a private and unending landscape of tall, wide trees, vines and the cacophany of tropical birds. She had been visiting in her dreams since she was a child, and now she was very familiar with it. She walked through the jungle unafraid and playful, holding out her arms for the birds that came to perch on her elbow. The jungle was endless and it never changed, except - except - who was that? A boy rather older than her walked through the jungle ahead of her, and he jumped when she called out in excitement. 'Who are you - what's your name?' 'Why should I tell you,' said the boy, 'since you are only a dream in my head?' ”

Topic: open seas, Mean novelty index: 6.05

There once was a pirate ship that set sail on the high seas to hunt for a great treasure. There was something special about this ship and her crew though and that was it was crewed by only monkeys but captained by a snake! Of course as you can imagine this mix of crew did not bode

well for the success of their mission. The trouble started as soon as they left port because of course the crew had no idea how to operate the ship! The captain being a snake could only hiss his instructions which the monkeys did not understand and they continued to just play around and eat all the supplies. After a few weeks of sailing aimlessly the ship ran a ground on a deserted island. Fortunately for the monkeys and the snake there was plenty of food and water for them on this island so they decided to make this their home. And this is how what we know now as the country called England was started.

Topic: open seas, Mean novelty index: 6.11

Becky knew she wasn't supposed to take her father's boat out of the harbour onto the open sea by herself, but it was a lovely day and the sea was still and calm, she'd watched her father operate the boat more times than she could remember so she felt confident as she started the motor and headed out.   Becky turned her face upwards towards the warm sun and closed her eyes, blissfully enjoying the heat and the sound of the water lapping at the sides of the boat. She felt a shadow pass overhead and opened her eyes, delighted to see the seagull flying over her head and the three others who had joined their brother, she felt giddy as they circled above her with their shrieking cries. Suddenly one of the gulls swooped lower and she felt a stabbing pain in her head as it's beak connected with it, another beak flew into her face and she screamed as she felt her flesh tear. Beak after razor-sharp beak pecked at her head and slashed her arms as she tried to protect herself. Blood was running down her face into her eyes and she could no longer see.   She curled into a ball at the bottom of the boat and waited for the onslaught to stop, and she waited and she waited and she waited. By mid-afternoon the next day an empty boat was seen gently drifting into the harbour.

Section 6.2. Human only, Median mean novelty

Topic: different planet, Mean novelty index: 3.97

In our adventure to another planet we left Earth with great intrepidation. Never before had anyone from Earth embarked on such an adventure. The journey will take around 4 1/2 years and in that time me and the rest of the crew shall spend alot of time together. I have always been fascinated by space travel and I cannot believe the day has come when I will experince this. On arriving at the planet we slowly tocuh down on the surface of the planet. As the door opens we wonder what we will face. My heart is racing as the door opens. There in front of us is an alien!

Topic: different planet, Mean novelty index: 4.00

She sat back in the padded chair, terrified; the countdown to launch was about to begin. Ten to-one came and went and she couldn't believe the feeling of weightlessness as the rocket sped to her new home: Gilga-3. Once arriving, she was welcomed by those who had flown there before her and was shown to her dormitory. The first day was exciting and surreal and she was shown around by a teenage boy who pointed out all the wildlife and points of interest they had currently found.   "Here is a Lonsha: It's like a rabbit but much smaller; and here is a Mount: It's like a

gerbil but much larger." The boy helped her around most of the day before giving her her daily duties for the following weeks. With her sharpshooting skills, she was to help the boy's dad hunt for food and explore some of the areas the citizens hadn't yet searched. The girl felt involved and well looked after here and at a first glance decided that this trip was the best thing she had ever done.

Topic: jungle, Mean novelty index: 4.00

Our story today begins in the deep undergrown of the Indian jungle. Jeremiah, an explorer, from Brooklyn New York, is in stealth mode, for he has just spotted a rare tiger. This, the sabretooth spotted tiger, is going extinct and Jeremiah needs to seek information of the breed to save them. Jeremiah has been studying them now for eight years, along with his girlfriend, June. However, did I mention that Jeremiah is in fact scared of tigers? Jeremiah doesn't know it yet, but he is brave, wise and very patient, the perfect concoction that the sabretooth spotted tigers need to survive. June knows this of course, but she isn't here yet to raise his morals. Let's see how our friend gets on.

Section 6.3. Human only, Lowest mean novelty

Topic: open seas, Mean novelty index: 1.54

I set out in my little rowing boat for an adventure on the seas. It was very cold so i wore a big coat and a hat. I also wore gloves to keep my hands warm. The sea was very choppy making my progress very slow. Despite rowing against the current i slowly moved forward. I rowed and rowed until i could see an island ahead. As i got closer to the island i dropped down a weight on a rope to hold the boat in one place. I then took out a rod and began to fish.

Topic: open seas, Mean novelty index: 1.67

Setting sail in a pirate vessel. Across the Indian ocean. What a beautiful sight. Steering the ship amid the hot temperatures. Admiring the wonderful clear sea. The sea is calm. We anchor just off the coast. And dive in the deep blue sea.

Topic: jungle, Mean novelty index: 1.94

There lived a man called George. George was born and lost in the jungle where he was brought up by monkeys. George lived an adventurous life playing and jumping with monkeys. when he reached the age of 18, he stumbled accross a lady called Fiona whose father and his men came to the jungle to capture animals. George fell in love with fiona. Fiona's father was against the love between his daughter and george. At the end, George got married to fiona after much struggle. Fiona,s father finally blessed their union.

Section 6.4. Human with 1 GenAI idea, Highest mean novelty

Topic: different planet, Mean novelty index: 5.94

A married couple and two cats decided to visit another planet. They researched the opportunities online and found the company which would take them to Saturn. Their preparations began by trying to float in the swimming pool and trying to hang upside down. They were just guessing the skills they would need to be okay on that journey and on that planet. The husband and wife were still waiting to receive the instructions from the company, but were both thrilled and nervous. The journey was about to happen in 1 month, but they were already telling their cats all about it. This adventure would be the only thing that would matter over the next weeks. Even the cats seemed to be excited!

Topic: different planet, Mean novelty index: 6.03

On a planet called Gwappy there lived a land dolphin called Gwoimpy. Being a land dolphin, Gwoimpy could walk, obviously, though not gracefully. Sometimes other animals would snicker when Gwoimpy shuffled by, but Gwoimpy honestly didn't care. He was long in the tooth, so to speak, and had seen it all. However, even though he wasn't offended in any way by their sleight, he did feel that rudeness should be punished, for how else would they learn? So one bright and sunny day, Gwoimpy headed off to where he knew the snickerers would probably be, and as he was shuffling ungainly past them, he pulled out the machine gun that he kept in his large backpack, and sprayed them all with lead. They all died. Gwoimpy, the psychopathic land dolphin on Gwappy, lived happily ever after.

Topic: different planet, Mean novelty index: 6.19

A crew of divers, several of the best in the world are determined to plot the depths of the oceans of Venus. Though designated as a crew, these group of divers hate each other and are nothing like a crew in reality. The researchers knowing this, design the submarine to be exactly manned by the 8 divers. If one person dies (or is killed) the submarine would become inoperable. While the rest of the divers are fine with this clause, there exists a mad man amongst them. Who is he? Nobody knows. Join us in this murder whodunnit set in the depths of space, where the twist is each person does their best to make sure everyone comes out alive against the whims of a mad man.

Section 6.5. Human with 1 GenAI idea, Median mean novelty

Topic: open seas, Mean novelty index: 3.95

A group of about 50 students from a college went on an adventurous open sea trip. They planned to go surfing in the ocean. Most of the students started to surf and some of them went a bit deeper in to the ocean and suddenly one of the student saw a shark and shouted. Everybody was terrified and started to surf back to the shore. But there were a few students who slipped

and fell into the ocean. They were terrified to the core that they didn't know what to do. One of the students who fell into the water began to sink and was helped by others to safety. At last it was known that there was no shark in the water and it was a prank by one of the student.

Topic: jungle, Mean novelty index: 4.00

A group of friends decided to go on a daring jungle adventure to find a missing airplane that crashed that contained many mysteries. The journey through the jungle led to many different experiences. One person struggled with food poisoning whilst another person had a twisted ankle from tripping over. Despite this they all still ventured deeper and deeper. As the friends got closer they heard weird noises coming from the area where the plane crashed. Slowly they got closer and closer. When they got to the aircraft they were shocked at what they saw. The friends haven't been heard from since.

Topic: open seas, Mean novelty index: 4.00

In search of an ancient treasure, a mighty crew embarks on a thrilling adventure across the Indian Ocean, battling treacherous storms and skilled pirates. As they navigate mysterious islands and decode cryptic symbols, their bond and loyalty begin to be tested by greed and deception. The captain is aware some of his crew are not on the ship for the thrill of the adventure, but rather for the potential wealth they might accrue from the treasure. On one dark night, the ship is attacked by a group of pirates, and the crew rises to the occasion. Forgetting their differences, everybody fights tooth and nail for the survival of their ship. The team emerges victorious, and in taking over the pirates' ship, they discover additional maps that could help them reach the treasure. Decoding the newfound clues, they are overwhelmed with joy and their ability to work together grows even more robust. Ultimately, they discover the true meaning of friendship and claim both treasures, the material one and the legacy they left behind that would inspire generations to come.

Section 6.6. Human with 1 GenAI idea, Lowest mean novelty

Topic: open seas, Mean novelty index: 1.79

It was a wonderful sunny day on the south coast. Paul and his friend decided to go out to sea in a boat. The journey started of well, on a nice calm ocean. But as they day went on, the weather took a turn. It started to get windy. The boat began to rock, and water was entering the boat. Paul tried to turn the boat to steer it back to shore. But it was extremely difficult in the wind. Just as it started to turn, the wind dropped and the sun came out again and all was calm at sea

Topic: open seas, Mean novelty index: 2.10

The young lads embarked on a adventure onboard their boat. They came across a storm soon after. They stayed afloat with luck on their side. The day after they saw an uninhabitated island where they landed. They explored this mysterious island of beauty. They will remember this

adventure in to their old age and reminice. The experience will prepare for more adventures in their lives. This will be a good start to more adventures to follow.

Topic: jungle, Mean novelty index: 2.27

Five school boys are about to have the best time of their lives.They have all agreed to go on an adventure in the jungle. They are accompanied by a group of teachers. The five school boys are looking for animals not familiar to the. They come across lots of snakes and spiders. Also there are monkeys swinging from trees. Their are rangers that patrol the outer bits of the jungle to keep everyone safe.The boys jump in the rangers van to explore deeper in the jungle ,but in a safe space.

Section 6.7. Human with 5 GenAI ideas, Highest mean novelty

Topic: different planet, Mean novelty index: 5.76

The generation starship went into orbit around Theta, the first new planet to be visited by humans.It had been travelling from Earth for 2000 years and now Jake was ready to travel to the surface in the lander craft.After landing he got out with his crew of eight others and looked around at the surface structures which looked exotic,just like pictures of rain forests once found on Earth.The trees nearby swayed and shifted in the light breeze and Jake saw the first alien life form ever seen .The creature had a round head but with lots of small eyes which covered the top half and the rest of the body looked more like a beetle.It crept toward the crew who looked at Jake asking with their gazes what they should do.He stepped forward slowly and approached the alien who held out its arm like structure to him.He held out his own arm in greeting and touched the alien but suddenly the rest of the crew saw them both disappear from view and did not know what to do next.

Topic: jungle, Mean novelty index: 5.87

The village was like a timewarp - Like looking into the past, but with an unusual twist. Everything was different. Houses where upside down, with the roof at the bottom and the door at the top, with no clear or visible way to get in. The villagers were friendly, but we could not understand them, nor they us. They were fascinated by us, our style, our equipment, and our overall look. With nowhere for us to stay, we tried to leave, but could not. Every time we left through the trees, via the same way we arrived, we ended up in the village again. We quickly realised that all was not as it seemed, and upon nightfall, things became very different.

Topic: jungle, Mean novelty index: 6.56

Florence would not say that she enjoyed nature exactly, but she kind of liked being alone in the jungle. Or at least, she thought she was alone. Wearing this headset made it difficult to appreciate what was real and what was not. Sounds suddenly invading her consciousness and brief, wraith-like images flickering at the edge of her vision. What here is imagined and what is

real; how much of what she was experiencing was she herself generating? She would not know for sure for another 59 minutes and eight seconds...

Section 6.8. Human with 5 GenAI ideas, Median mean novelty

Topic: open seas, Mean novelty index: 3.97

After losing their fishing boat in a merciless storm, John Anderson and his dog, Buster, embark on an unexpected journey surviving on a life raft in the middle of the Pacific Ocean. They encounter ruthless pirates, large marine animals and endure harsh weather conditions. In their quest to survive, they discover an uncharted island guarded only by an old wickery gate. John battles the crashing waves and beaches his raft, he leaps off with Buster and explores the small abandoned island when he suddenly sees a glowing chest. His eyes bulge with excitement and he runs over until suddenly a group of pirates appear out of nowhere and start attacking John. John with the help of Buster and his old pistol defeat the pirates without even suffering a scratch! He walks over to the glowing chest and opens it, it flips back and the piercing light of treasure lights up the island. John and Buster have done it, he attaches rope to the chest and pulls it back to his raft.

Topic: open seas, Mean novelty index: 4.00

The three teens had been on an adventure course the previous week and to this end had found themselves embarking on a voyage in a small dinghy from a private jetty belonging to the father of one of them (Raffie). It was after midnight and the moon lit them up as they pushed off excitedly. Shap, who was the natural leader looked at his compass confidently and steered north west, not noticing something brushing his oar lightly. After 20 minutes or so, a fin appeared alongside them, dipping occasionally. Noticing it, Lara, the youngest, clutched at her brother Finn, nearly knocking him out of the boat in fright. The panic was contagious as everyone started standing up and knocking into each other, pointing and shouting. Raffie and Shap quickly turned the boat and began to head for shore but the fin turned with them and followed ominously. Picking up the pace, the boys paddled furiously and made for shore as fast as they could.

Topic: open seas, Mean novelty index: 4.03

I was born on the south coast and everyday i watched boats go out to sea. I always wanted to go on one but it seemed that I would never have the chance. But one day a friend asked me if I would like to join him on a trip to France. I was very excited, and said Yes. We planned the trip carefully, or so we thought, and left early one morning. However. strong winds got up and blew us onto a sandbank. We had to radio for help and eventually we were rescued and towed back to land.

Section 6.9. Human with 5 GenAI ideas, Lowest mean novelty

Topic: open seas, Mean novelty index: 1.92

One day i sailed with a boat into the deep blue sea. I saw a shark and panicked. I tried to sail back but my daughter was fascinated with the shark. So i waited for some time to see if the shark would circle back to the island. We did not have much food only a few crackers. My daughter was so small she started throwing crackers into the sea. I panicked even more thinking the shark would come back to eat us. After some time the shark disappeared we were relieved and sailed back to the island.

Topic: jungle, Mean novelty index: 2.61

Ice was added to a cup.   Then lemonade was added to the cup. The cup was carried into another room. Then the cup was then placed on a coaster on a table. Then the cup was picked up but slipped and feel to the floor.   The cup shattered and broke into many pieces. The lemonade and ice was spilt all over the floor. A nearby cloth was used to wipe up the mess caused by the cup being spilt.

Topic: open seas, Mean novelty index: 2.64

It was the first day of summer holidays.Jack and Sophie couldn't part away and decided to spend this lovely Monday together.Walking along the seashore they suddenly noticed something in the bushes.There was an really old boat. It condition indicated that it had been there for a long time. Friends come closer and decided to sail because the overall condition of boat was satisfying. They didn't notice the small hole in the middle of the deck.This day had a very sad ending .

Section 6.10. Human only, Highest mean usefulness

Topic: open seas, Mean usefulness index: 6.79

Luella struggled to stay upright as the strong gusts of wind blew against her and the boat suddenly swayed beneath her. When she set out for a new life in America, little did she imagine that it would be a fight for survival to get there. Tired of having barely enough food to stop her stomach cramping in pain at night, Luella had eagerly grabbed the opportunity to become a governess to the two young children of the local squire. The children - where were the children now? Luella realised that she had been so focused on keeping her balance that she had stopped watching them. Looking across the misty deck she could see one of her young charges was sheltering beneath a seat but the other? She saw young Amelia desperately trying to hang on to the railings and not be swept over. With a sudden burst of energy, Luella rushed forward and grabbed the young girl's dress, pulling her violently away from the edge just before the next strong gust.

Topic: jungle, Mean usefulness index: 6.89

Will and Emma's boat crashed ashore, breaking into hundreds of pieces as it smashed against the rocks. They had gone off course during their round the world challenge, had no idea where they were, and beyond the small, rocky beach they had touched down on, all they could see in both directions was a wall of tall, thick trees. It was beginning to get dark and they needed to seek help, if not, shelter. Will and Emma entered the jungle, holding each others' hands tightly, and after a few minutes of walking, a bright light started to appear from among the vine-covered trees. They headed towards the light, despite being unsure of what it was - they hoped it would be a place of sanctuary where they would find help or, at the very least, a place to stay the night. As they edged closer, many sounds echoed around them - buzzes, roars, and the screams of primates. Will and Emma picked up their pace until they were face to face with what was creating the light, where they found, to their surprise, nothing but an ancient, freestanding door. Should they open it?

Topic: open seas, Mean usefulness index: 7.00

Kye peered over the edge of the boat and shuddered. The thick mass of water undulated below him, dark and impenetrable. They still had 600 miles before reaching Hamlet Cove, and the way was looking grim. Up ahead was a wall of grey cloud, and following them from the South, the same. The ship was quiet that day. They all knew what challenges lay ahead, and an unnerving presence hung in the air. Kye turned to the captain. He had never seen him look so pale.

Section 6.11. Human only, Median mean usefulness

Topic: different planet, Mean usefulness index: 5.33

Landing on the planet Huypto began during the sunset which is more beautiful than can ever be imagined. This place can only be described as a jungle of colour, it is a place you would see in the films made purely from animation. Life on Huypto is more peaceful and relaxing to that on earth with creatures that are not fixated on pray. Food is existing naturally with vibrant fruits similar to dragonfruit. Being a carnivore here seems a concept that would have never existed. I begin my first few hours on Huypto by exploring the surroundings and picking somewhere that myself and my colleagues will make as our base. It is seeming to be more of a holiday than an exploration job so far. My view from my tipi is like a picture, I never want to leave this place!

Topic: different planet, Mean usefulness index: 5.36

On the planet zoom there lived a ginger haired gnome queen called Lola and ruled over the planet zoom with her trusty sidekick half cat half alien called charlie. Charlie roamed the land searching for invaders and traitors to Lola. Lola would cast spells on anyone or anything that caused harm to her loyal subjects which included differnet species from othe planets who had escaped persecution or the risk of death on there home planet, and they were made to feel welcome and cared for on planet zoom. Lola encouraged the new comers to intigrate into the community and to aslo embrace there own traditons and beliefs. This worked extremely well

and the locals loved learning about different planets and traditions they had never seen before. One day charlie caught invaders from another planet trying to take back some of their people who had fled their home planet. Charlie took the to the queen who turned them all in miniature gnomes and made the help all the newcomers on the planet as punishment. Lola continued as queen for years and made sure all her subjects helped each other, and made all her subjects aware of how important being kind and excepting of different cultures and traditions regardless of where you come from.

Topic: jungle, Mean usefulness index: 5.36

Rachel and Emily crouched to look at the tiny, dark spider. 'Careful Rach,' Emily warned, resting her hand on her sister's. 'That might be venomous.' 'But Mrs Webb told us to find the most interesting thing we could.' Emily stood up, stretching her back as she looked up at the high canopy of the trees and then around at the shadowy forest floor. They'd wandered quite a way from the college group and she couldn't hear anyone else, or anything else for that matter. 'Do you remember the way back Rach?' 'We just need to follow the string you teased me for bringing,' Rachel said with a laugh.

Section 6.12. Human only, Lowest mean usefulness

Topic: open seas, Mean usefulness index: 2.58

visited a cage,the entrance was so low. with some beautiful trees around it. we went into it along with the cave guards. it was dark, we made use of torchlight. the view was so beautiful and astonishing. we heard noises of bats. we moved from one section of the cave to another. certain sections we had to craw to get in . the experience was very exciting

Topic: opens seas, Mean usefulness index: 2.94

Today we are leaving for a week adventure on a private boat on the open sea, everyone is very exited and looking forward to the trip. I will be going with my partner and friend and their partner. We have all packed and ready to leave, we get to the boat and get on board we set off into the ocean the sea is a little choppy at first but then it calms down. All of the sudden we see dolphins in the water along side the boat it was very exiting to see them. The evening arrives and we make dinner on the boat we had salad with prawns. The next few days go by and the waether has been great it has been very relaxing. On the last day we decide to go by a island to have a look it looks like no one lives there but is a beautiful place. Its the final day and we make our way home after a great week away on the open sea.

Topic: jungle, Mean usefulness index: 3.06

In a hot and humid summer, our family decided to go on a jungle holiday. We are very excited and really looking forward to this adventure together. We landed and met our guide at the airport. Our adventure begins. He called John and he is very experienced about this land. He

took us to the jungle in his jeep which was very exciting.   We saw a herd of buffalos just chilling out.   We also saw some zebras.

Section 6.13. Human with 1 GenAI idea, Highest mean usefulness

Topic: different planet, Mean usefulness index: 7.21

Three bold, adventurous astronauts departed Earth on a mission to uncover life on a newly discovered planet. They left behind everything they'd ever known with no idea when or if they would return, but they did it in the name of science. The journey was long and arduous, but they hoped everything they discovered would make it worthwhile. As they arrived on the small, rocky planet, they realised just how far away they were from home. Everything looked strange and new, and they felt a deep sense of unease as they considered that not every creature in this world would be friendly. As they began to explore, they uncovered strange landscapes, astonishing lifeforms and ancient ruins, and it isn't long before they stumble across a powerful secret that will forever alter their perception of the universe. The astronauts must now choose whether to share their revelation with the world or keep the planet's mysteries for themselves. The captain sits down in front of the monitor, their only connection to planet EartH, and makes his decision.

Topic: open seas, Mean usefulness index: 7.36

The captain stared out from his position on the bridge. The sea was slate grey and looked angry, he thought. The wind was whipping up and the captain pulled his collar up to shelter from the biting cold. He had been on plenty missions like this before, but this was a different proposition altogether. At first, he had thought the President must have been mistaken, and that this was a wild goose chase. But as he peered through his binoculars, he realised that the intelligence had been correct. After a thousand years and countless more stories and theories, he was looking straight at it. Atlantis.

Topic: different planet, Mean usefulness index: 7.58

The year is 2123 and over-consumption of Earth's natural resources has created a barren landscape in rural areas, with most of Earth's population flocking to large cities where the few remaining resources are stockpiled. Dwindling traditional forms of energy generation, such as oil, coal, and water, has created a desperate need to find an alternative energy source to power these gargantuan metropolises. In a daring mission to solve this growing issue a group of fearless astronauts embark on an unpredictable adventure to the planet Xyrus. Battling extraterrestrial creatures, overcoming harsh climates, and deciphering strange symbols to unlock ancient secrets, they discover a powerful energy source that could change life as we know it. The team stoically battle their way across the planet, back to their ship, facing staunch resistance from the native Xyrian population. They reach their ship just ahead of the Xyrian defenders, but there isn't enough time for the team to board and take-off before they are

overrun. In a last ditch attempt to ensure the success of the mission, the team's commander, Mac, hangs back and sacrifices himself to give the rest of team a chance of survival. Due to Mac's heroism, the remaining team manage to take-off and, after a long journey through the vast expanse of space, they arrive back on Earth to find the technologically superior Xyrian ships waiting for them!

Section 6.14. Human with 1 GenAI idea, Median mean usefulness

Topic: jungle, Mean usefulness index: 5.33

The adventure began here, I was wondering into the jungle, in search of something treasured, something valuable something no-one had ever come across before, the emerald egg. I had been on my hunt for many years now but all the clues lead here, to this very jungle. It wasnt going to be easy though. I trenched the jungle for days, my shoes became wet from the rainfall, I smelt and I was growing tired, especially after being on the lookout for dangerous animals. I was growing very tired, but my determination was strong, I had been through so much to be here, I was going to prove the non believers wrong and I was going to be rich too. I was thinking of setting up camp soon, but thats when I heard sounds in the distance. It sounded like humans, but it wasn't in any language I understood, but I decided to make my way over to the sounds. When I reached the destination, thats when I saw it the people, were crowded but in the middle of them the chief was holding the emerald egg!

Topic: different planet, Mean usefulness index: 5.33

On arrival, Fazziel and the others had been astonished by the uncanny strangeness of the sky with its odd green tinge, and the three suns. The interweaving cycles of Short Day and Long Day and Night had played havoc with their sleep patterns. Without the welcoming, gentle diplomacy of the Blue People. they were sure they would have fallen foul of the more belligerent inhabitants, in their weakened and confused state. As things had progressed toward what they now thought of as the "new normal" of life on the planet, it gradually, though slowly, became apparent that there were complications in the relations between the different sentient species, which they would never fully understand, and must be very careful to tread around. This, of course, is to be expected in all first contact scenarios, and was a major focus of their training, but it is recognised that the particular nuances of a given situation can never be foreseen, or fully comprehended, Caution and humility are absolutely crucial, and yet, in spite of meticulous training and selection of participants, unpredictable difficulties always arise. Thus, I could not be surprised to learn of the perilous conditions which had developed over the course of that first World-Year (which I must remind you, is equivalent to almost three Earth-years); on the contrary, I must commend the unit for the tenacity and astuteness with which they were able to navigate the relationships, such that there is now a flourishing and mutually beneficial alliance between the parties. The peaceful conditions which now prevail have enabled the unit to pursue their research and develop a broader knowledge of the various inhabitants, which I shall describe in as much detail as possible in this report.

Topic: jungle, Mean usefulness index: 5.39

An explorer embarks on a journey through an uncharted jungle, confident and curious, they have a desire to uncover its hidden mysteries. It is told there is all sorts of undiscovered finds in the massive jungle and the explorer intends to unravel it. Along the way, they forge unexpected alliance with the diverse creatures inhabiting the tropical landscape and uncover ancient ruins filled with lost treasures and ornaments. The explorer enjoyed the company of apes, snakes, and other unlikely strangers. It is a story that is unlikely to be believed. Through perseverance, the explorer ultimately uncovers the secrets of the jungle and returns home with tales of adventure, inspiring others to seek out their own extraordinary experiences. Maybe one day others will be with the animals too. Maybe one day, they will feel fulfilled.

Section 6.15. Human with 1 GenAI idea, Lowest mean usefulness

Topic: jungle, Mean usefulness index: 2.47

a man and a woman are in the jungle. there are there to study the lacal wildlife. one day they find a type of mushroom that has never been seen before. they pic, and pacage up a few samples to take back with them. after spending a few more days in the jungle they head back home. when they get home they begign to study the new mushroom. it is discouvered that the mushroom has great health beinfits. they become rich and famous from thier discovery.

Topic: different planet, Mean usefulness index: 2.78

an astronaut heads off to a distant planet.he does not know if it is inhabited as it is obscured by cloud.when he touches down safely he is relieved.on exploring he finds no signs of life,further exploration reveals tunnels under the surface.he enters them only to find they are empty.there is no life.reluctantly he reboards his ship & leaves.on returning to earth he reports his findings.

Topic: jungle, Mean usefulness index: 3.00

once upon a time, two friends set out for a thrilling adventure in a jungle. there they found a lost ancient temple. Inside the temple was lost treasure that possess supernatural powers. the friends must learn new trick and pass all puzzles to be able to access the treasure and take it home. one of the friend was calm and start learning the language with which the temple instructions were written. The other was going around checkng if there was a back door to the treasure. luckily the friend learned the language and passed all puzzles. they finally got the treasure and went home happy.

Section 6.16. Human with 5 GenAI ideas, Highest mean usefulness

Topic: jungle, Mean usefulness index: 6.80

The village was like a timewarp - Like looking into the past, but with an unusual twist. Everything was different. Houses where upside down, with the roof at the bottom and the door at the top, with no clear or visible way to get in. The villagers were friendly, but we could not understand them, nor they us. They were fascinated by us, our style, our equipment, and our overall look. With nowhere for us to stay, we tried to leave, but could not. Every time we left through the trees, via the same way we arrived, we ended up in the village again. We quickly realised that all was not as it seemed, and upon nightfall, things became very different.

Topic: different planet, Mean usefulness index: 7.11

Susan gazed at the foreign landscape, her heart pounding with excitement. The air crackled with unfamiliar energy, and the vibrant colors of the alien flora mesmerized her. She took a hesitant step forward, her boot stinking into the lush, emerald grass. As she explored further, strange creatures darted through the trees, their iridescent wings shimmering in the sunlight. A gentle breeze carried the scent of exotic flowers, filling her senses. The sky above revealed celestial wonders unknown to Earth, with heavenly bodies dancing in a cosmic ballet. Susan's heart swelled with a sense of wonder and adventure, as she realized she was the first human to set foot on this extraordinary planet. At that moment, she knew she had embarked on a journey that would change her life forever, one filled with discovery, challenges, and the limitless possibilities of the universe.

Topic: jungle, Mean usefulness index: 7.67

Florence would not say that she enjoyed nature exactly, but she kind of liked being alone in the jungle. Or at least, she thought she was alone. Wearing this headset made it difficult to appreciate what was real and what was not. Sounds suddenly invading her consciousness and brief, wraith-like images flickering at the edge of her vision. What here is imagined and what is real; how much of what she was experiencing was she herself generating? She would not know for sure for another 59 minutes and eight seconds...

Section 6.17. Human with 5 GenAI ideas, Median mean usefulness

Topic: different planet, Mean usefulness index: 5.33

As human civilisation progresses to 2356. Advances are made in intergalactic spacecraft technology. A new planet is found for a lucky 6 rich businessmen. Planet x1 is green and vast with pools and jungles. Everything on the planet is edible. Its a magical world with time portals and teleportation black holes to each area of the planet. The exploration is at first treated as a

holiday and the businessmen enjoy there time exploring many wonders. But soon unbeknown to all the research disaster strikes! Out of caves walk aliens with with weapons to freeze enemy's and terrorise

Topic: open seas, Mean usefulness index: 5.38

I was born on the south coast and everyday i watched boats go out to sea. I always wanted to go on one but it seemed that I would never have the chance. But one day a friend asked me if I would like to join him on a trip to France. I was very excited, and said Yes. We planned the trip carefully, or so we thought, and left early one morning. However. strong winds got up and blew us onto a sandbank. We had to radio for help and eventually we were rescued and towed back to land.

Topic: jungle, Mean usefulness index: 5.39

Once upon a time there was a brave young girl. One day, she decided it was time for an adventure. She gathered a group of friends and embarked deep into the heart of the jungle, on a thrilling journey to find a lost city rumoured to hold ancient treasures. Together they encountered venomous snakes, deceptive quicksand, and hostile native tribes. Relying on each other for support, they were courageous and determined, and eventually came upon the legendary lost city. As they explored the city, they came to realise the true treasure was their journey of self-discovery and the friendships they built along the way. Of course, they also located the ACTUAL treasure and decided to split it amongst themselves equally. They each took their share home and lived happily ever after.

Section 6.18. Human with 5 GenAI ideas, Lowest mean usefulness

Topic: jungle, Mean usefulness index: 1.39

Ice was added to a cup.   Then lemonade was added to the cup. The cup was carried into another room. Then the cup was then placed on a coaster on a table. Then the cup was picked up but slipped and feel to the floor.   The cup shattered and broke into many pieces. The lemonade and ice was spilt all over the floor. A nearby cloth was used to wipe up the mess caused by the cup being spilt.

Topic: open seas, Mean usefulness index: 2.47

One day i sailed with a boat into the deep blue sea. I saw a shark and panicked. I tried to sail back but my daughter was fascinated with the shark. So i waited for some time to see if the shark would circle back to the island. We did not have much food only a few crackers. My daughter was so small she started throwing crackers into the sea. I panicked even more thinking the shark would come back to eat us. After some time the shark disappeared we were relieved and sailed back to the island.

Topic: different planet, Mean usefulness index: 3.15

Author had not seen his old friends since finishing astronaut training almost seven years ago, he was delighted when they all said they would come for a belated Birthday weekend to visit the newly built casino city of sin province on Mars. Usually Author will partake in a few shots of Martian liquor when he is frying the space ship with no consequences. Unfortunately he is usually flying alone with no distractions. On this occasion there was plenty of distractions. In the celebrations of Authors belated Birthday and his 4 oldest friends and the Martian liquor flowing a catastrophe was looming. Author saw the lights below of the city of sin and lowered the ship not realizing exactly how close those lights really were until he smashed through the front of the newly built Martian Palace Casino. The glass front was completely collapsed, Author and his friends were unhurt physically but there was much damaged pride, the Martian security however were so angry they were foaming at the mouth. Author and his friends spent the belated Birthday weekend in a Martian cold cell nursing a hangover, they all agreed next year to just send a card.

Section 7. Writer study screenshots

Study Overview

Overview: This study will consist of two parts and a short follow-up survey. In some parts, you will be asked understanding questions. You must answer these understanding questions correctly in order to proceed to complete the study.

Payment: For completing this study, you are guaranteed to receive a £3.00 within 48 hours. In addition, one part of the two parts will be randomly selected as the part-that-counts. Any amount (if any) you earn in the part-that-counts will be distributed to you as a bonus payment after 4-6 weeks.

Understanding Question: Which of the following statements is true?

- ○ For completing this study, I will receive £3 within 48 hours, but I do NOT have a chance of receiving any additional bonus payment.
- ○ For completing this study, I will receive £3 within 48 hours, and I will also receive the amount I earn in the part-that-counts as additional bonus payment
- ○ For completing this study, I will receive £3 within 48 hours, and I will also receive the total amount I earn across all parts as additional bonus payment.

Next

Part 1

Instructions

Please enter 10 words that are as **different** from each other as possible, in all meanings and uses of the words.

Rules

- Only **single words** in English.
- Only **nouns** (e.g., things, objects, concepts).
- **No proper nouns** (e.g., no specific people or places).
- **No specialised vocabulary** (e.g., no technical terms).
- Think of the words **on your own** (e.g., do not just look at objects in your surroundings).

Enter words

1.

2.

3.

4.

5.

6.

7.

8.

Condition: *Human only*

Part 2

Instructions

We would like you to write a story about **an adventure in the jungle**. You can write about anything you like. The story must be **exactly eight sentences long** and it needs to be written in English and appropriate for a **teenage and young adult audience** (approximately 15 to 24 years of age).

Please write your story (**exactly 8 sentences**) here:

Current sentence count: **0**

You need to write 8 sentences to enable the Next button.

Sentences end with a full stop (.) a question mark (?) or an exclamation mark (!) Next

Condition: *Human with 1 AI idea* (before story has been generated)

Part 2

Instructions

We would like you to write a story about **an adventure in the jungle**. You can write about anything you like. The story must be **exactly eight sentences long** and it needs to be written in English and appropriate for a **teenage and young adult audience** (approximately 15 to 24 years of age).

In order to assist you, we have provided you **access to AI assistance** that, if you wish, will come up with a starting point for your story by clicking on **"Generate Story Idea"**. The response from the AI assistant will be created in real time by a sophisticated AI algorithm. The response will be added in grey text below. **You are free to use or disregard any element of the AI assistant's story idea, or start over with your own idea**.

Generate Story Idea...

Please write your story (**exactly 8 sentences**) here:

Current sentence count: **0**

You need to write 8 sentences to enable the Next button.

Sentences end with a full stop (.) a question mark (?) or an exclamation mark (!) Next

Condition: *Human with 1 AI idea* (after story has been generated)

Part 2

Instructions

We would like you to write a story about **an adventure in the jungle**. You can write about anything you like. The story must be **exactly eight sentences long** and it needs to be written in English and appropriate for a **teenage and young adult audience** (approximately 15 to 24 years of age).

In order to assist you, we have provided you **access to AI assistance** that, if you wish, will come up with a starting point for your story by clicking on **"Generate Story Idea"**. The response from the AI assistant will be created in real time by a sophisticated AI algorithm. The response will be added in grey text below. **You are free to use or disregard any element of the AI assistant's story idea, or start over with your own idea**.

Three friends embark on a thrilling adventure in the unexplored depths of the Amazon rainforest in search of a legendary ancient city. Battling unpredictable weather, dangerous wildlife, and treacherous terrains, they uncover hidden tribal mysteries and ancient secrets. Their friendship and courage are tested to the limits as they face life-threatening challenges, leading to a climax that changes their lives forever.

Please write your story (**exactly 8 sentences**) here:

Current sentence count: **0**

You need to write 8 sentences to enable the Next button.

Sentences end with a full stop (.) a question mark (?) or an exclamation mark (!) Next

Condition: *Human with 5 AI ideas* (before story has been generated)

Part 2

Instructions

We would like you to write a story about **an adventure in the jungle**. You can write about anything you like. The story must be **exactly eight sentences long** and it needs to be written in English and appropriate for a **teenage and young adult audience** (approximately 15 to 24 years of age).

In order to assist you, we have provided you **access to AI assistance** that, if you wish, will come up with a starting point for your story by clicking on **"Generate Story Idea"**. The response from the AI assistant will be created in real time by a sophisticated AI algorithm. You may select each of the 5 tabs below, and click the "Generate Story Idea" button. The response will be added to that tab.

Here is a guide to the status of the AI request in each tab:

- Free You have not generated a story idea in this tab. Click to start generating a new story.
- Used The AI has provided you with a suggested starting point for your story. Click to view the AI's suggestion.
- Error Sometimes the AI is busy and the request fails. Click to request a new story.

In each Used tab, the AI assistant will come up with another story idea that will be added to the list of starting points for your story. **You are free to use or disregard any element of the AI assistant's story idea, or start over with your own idea.**

Idea 1 Free | Idea 2 Free | Idea 3 Free | Idea 4 Free | Idea 5 Free

Generate Story Idea...

Please write your story (**exactly 8 sentences**) here:

Current sentence count: **0**

You need to write 8 sentences to enable the Next button.

Sentences end with a full stop (.) a question mark (?) or an exclamation mark (!) Next

Condition: *Human with 5 AI ideas* (after story has been generated)

Part 2

Instructions

We would like you to write a story about **an adventure in the jungle**. You can write about anything you like. The story must be **exactly eight sentences long** and it needs to be written in English and appropriate for a **teenage and young adult audience** (approximately 15 to 24 years of age).

In order to assist you, we have provided you **access to AI assistance** that, if you wish, will come up with a starting point for your story by clicking on **"Generate Story Idea"**. The response from the AI assistant will be created in real time by a sophisticated AI algorithm. You may select each of the 5 tabs below, and click the "Generate Story Idea" button. The response will be added to that tab.

Here is a guide to the status of the AI request in each tab:

Free You have not generated a story idea in this tab. Click to start generating a new story.

Used The AI has provided you with a suggested starting point for your story. Click to view the AI's suggestion.

Error Sometimes the AI is busy and the request fails. Click to request a new story.

In each Used tab, the AI assistant will come up with another story idea that will be added to the list of starting points for your story. **You are free to use or disregard any element of the AI assistant's story idea, or start over with your own idea.**

Idea 1 Used | Idea 2 Used | Idea 3 Free | Idea 4 Free | Idea 5 Free

While on a research expedition in the Amazon, a young scientist, Ava, discovers an ancient, mystical artifact in a hidden temple. The artifact's removal awakens a protective spirit, and Ava must navigate treacherous terrain, wild beasts, and magical obstacles to return it and calm the enraged spirit. Along her journey, she learns important lessons about respect for nature and ancient cultures, eventually succeeding in her quest and forging a profound connection with the Amazon.

Please write your story (**exactly 8 sentences**) here:

Current sentence count: **0**

You need to write 8 sentences to enable the Next button.

Sentences end with a full stop (.) a question mark (?) or an exclamation mark (!) Next

Follow-up survey

We have a few questions about your experience today. You will need to complete all of the questions in order to receive your payment

Next

Only shown in condition: *Human only*

Follow-up survey

Please tell us whether you used ChatGPT or a similar generative AI tool to inspire your story? (Please answer truthfully: your truthful answer will help us with our research. Your answer will NOT affect your payment.)

◯ Yes ◯ No

Next

Only shown in condition: *Human only* and answered “yes” on previous page

Follow-up survey

Here is the story you have submitted:

....

To what extent do you think did the AI generated assistance affect the story you have submitted?

(Please answer truthfully: your truthful answer will help us with our research. Your answer will NOT affect your payment.)

	Not at all<br>1	2	3	4	5	6	7	8	Extremely<br>9
**Please indicate to what extent the AI generated assistance affect the story you have submitted**	○	○	○	○	○	○	○	○	○

You will need to rate the AI generated idea on order to enable the Next button

Next

Follow-up survey

Here is the story you have submitted:

....

Please indicate you how much you agree with the following statements on the following scale: 1=Not at all, 5=Moderately, 9=Extremely:

	Not at all 1	2	3	4	5	6	7	8	Extremely 9
**This story has a surprising twist.**	○	○	○	○	○	○	○	○	○
**This story has changed what I expect of future stories I will read.**	○	○	○	○	○	○	○	○	○
**This story is funny.**	○	○	○	○	○	○	○	○	○
**This story is boring.**	○	○	○	○	○	○	○	○	○
**This story is well written.**	○	○	○	○	○	○	○	○	○
**I enjoyed writing this story.**	○	○	○	○	○	○	○	○	○

Next

Follow-up survey

Here is the story you have submitted:

....

	Not at all<br>1	2	3	4	5	6	7	8	Extremely<br>9
**To what extent do you think your story reflects your own ideas?**	○	○	○	○	○	○	○	○	○

Next

Follow-up survey

Here is the story you have submitted:

....

	Not at all<br>1	2	3	4	5	6	7	8	Extremely<br>9
**How novel do you think your story is?**	○	○	○	○	○	○	○	○	○
**How original do you think your story is?**	○	○	○	○	○	○	○	○	○
**How rare (e.g. unusual) do you think your story is?**	○	○	○	○	○	○	○	○	○

Next

Follow-up survey

Here is the story you have submitted:

....

	Not at all<br>1	2	3	4	5	6	7	8	Extremely<br>9
**How appropriate do you think is your story for the intended audience?**	○	○	○	○	○	○	○	○	○
**How feasible to do you think is your story to be developed into a complete book?**	○	○	○	○	○	○	○	○	○
**How likely do you think would it be that your story is turned into a complete book if a publisher read it and hired a professional author to expand on the idea?**	○	○	○	○	○	○	○	○	○

Next

Only shown in condition: *Human with 1 AI idea*

Follow-up survey

Here is the story you have submitted:

....

To what extent do you think did this specific AI generated story idea affect the story you have submitted?

(Please answer truthfully: your truthful answer will help us with our research. Your answer will NOT affect your payment.)

Three friends embark on a thrilling adventure in the unexplored depths of the Amazon rainforest in search of a legendary ancient city. Battling unpredictable weather, dangerous wildlife, and treacherous terrains, they uncover hidden tribal mysteries and ancient secrets. Their friendship and courage are tested to the limits as they face life-threatening challenges, leading to a climax that changes their lives forever.

	Not at all 1	2	3	4	5	6	7	8	Extremely 9
**Please indicate to what extent this specific AI generated idea affected the story you submitted**	○	○	○	○	○	○	○	○	○

You will need to rate the AI generated idea on order to enable the Next button

Next

Only shown in condition: *Human with 5 AI ideas* (before rating any story)

Follow-up survey

Here is the story you have submitted:

....

To what extent do you think did this specific AI generated story idea affect the story you have submitted?

Please respond to this question for each tab with a response that has the Rate me icon

(Please answer truthfully: your truthful answer will help us with our research. Your answer will NOT affect your payment.)

Idea 1 Rate me | Idea 2 Rate me | Idea 3 Unused | Idea 4 Unused | Idea 5 Unused

When siblings Ryan and Emily discover an ancient map in their grandpa's attic, they become captivated by the promise of unknown treasure and launch an unprecedented journey into the dense, dangerous jungle. The siblings overcome deadly obstacles, ally with indigenous tribes, and unearth secrets of their family's explorative past. Their adventurous spirit leads them to an unimaginable treasure, providing not only wealth but also an understanding of their family's courageous legacy.

	Not at all 1	2	3	4	5	6	7	8	Extremely 9
**Please indicate to what extent this specific AI generated idea affected the story you submitted**	○	○	○	○	○	○	○	○	○

You will need to rate all of the AI generated ideas on order to enable the Next button

Next

Only shown in condition: *Human with 5 AI ideas* (after rating a story)

Follow-up survey

Here is the story you have submitted:

....

To what extent do you think did this specific AI generated story idea affect the story you have submitted?

Please respond to this question for each tab with a response that has the Rate me icon

(Please answer truthfully: your truthful answer will help us with our research. Your answer will NOT affect your payment.)

Idea 1 ✓ Rated | Idea 2 Rate me | Idea 3 Unused | Idea 4 Unused | Idea 5 Unused

While on a research expedition in the Amazon, a young scientist, Ava, discovers an ancient, mystical artifact in a hidden temple. The artifact's removal awakens a protective spirit, and Ava must navigate treacherous terrain, wild beasts, and magical obstacles to return it and calm the enraged spirit. Along her journey, she learns important lessons about respect for nature and ancient cultures, eventually succeeding in her quest and forging a profound connection with the Amazon.

	Not at all 1	2	3	4	5	6	7	8	Extremely 9
**Please indicate to what extent this specific AI generated idea affected the story you submitted**	○	○	○	○	○	○	○	○	○

You will need to rate all of the AI generated ideas on order to enable the Next button

Next

Follow-up survey

Here is the story you have submitted:

....

In your own words, who do you identify as having provided the original spark and idea for this story?

Next

Follow-up survey

If this story were published and sold tomorrow, how much of the story's profit do you believe should belong to you versus other entities (such as prior books, stories, or AI tools) that may have provided the starting point for your story?

(Please answer truthfully: your truthful answer will help us with our research. Your answer will NOT affect your payment.)

Please indicate the percentage of the story's profit that you believe you should receive:

50%

0% 25% 50% 75% 100%

Next

Follow-up survey

	Not at all 1	2	3	4	5	6	7	8	Extremely 9
**How creative do you consider yourself?**	○	○	○	○	○	○	○	○	○
**How much creativity is required in your job?**	○	○	○	○	○	○	○	○	○
**How comfortable are you with new technologies?**	○	○	○	○	○	○	○	○	○
**How much (if at all) have you previously engaged with AI or similar technologies?**	○	○	○	○	○	○	○	○	○

Have you used any of the following AI tools in the past? (Check all that apply)

- ☐ None
- ☐ ChatGPT
- ☐ Dall-E
- ☐ OpenAI's playground (e.g. DaVinci, Currie, Ada)
- ☐ Stable Diffusion
- ☐ NightCafe
- ☐ Jasper
- ☐ Microsoft Bing Chat
- ☐ Google Bard
- ☐ You.com
- ☐ Midjourney
- ☐ Other

Ai Tools Other Name

____

Have you used any of the following categories of AI tools in the past? (Check all that apply)

- ☐ None
- ☐ Text
- ☐ Image
- ☐ Audio
- ☐ Music
- ☐ Video

Next

Demographics

What gender do you identify with?

- ○ Female
- ○ Male
- ○ Prefer not to say
- ○ Other (please specify below)

Other gender

[ ]

What is your current age? (enter a number of years)

[ ]

What is your highest level of education?

- ○ Less than A levels
- ○ Vocational training
- ○ A levels
- ○ Undergraduate degree
- ○ Postgraduate Master's degree
- ○ Professional degree (e.g. MBA, JD)
- ○ Doctorate

What is your current employment status?

- ○ Employed full time
- ○ Employed part time
- ○ Unemployed looking for work
- ○ Unemployed not looking for work
- ○ Retired
- ○ Student
- ○ Disabled

What is your current job title?

[ ]

What is your current annual income?

- ○ Less than £10,000
- ○ £10,000-£24,999
- ○ £25,000-£49,999
- ○ £50,000-£74,999
- ○ £75,000-£99,999
- ○ £100,000-£124,999
- ○ £125,000-£149,999
- ○ More than £150,00

Do you have any additional comments about this survey?

[ ]

Next

Section 8. Evaluator study screenshot

Study Overview

Overview: This study will consist of two main parts and a short follow-up survey. In each of the two main parts, you will be asked to read 6 short (eight-sentence) stories and answer a series of questions about them.

Payment: For completing this study, you are guaranteed to receive a £3 within 48 hours.

Please answer all questions carefully and honestly.

Understanding Question: Which of the following statements is true?

- ◯ For completing this study, I will receive £3 within 48 hours plus an unspecified bonus payment.
- ◯ For completing this study, I will receive £3 within 48 hours.
- ◯ For completing this study, I will not receive any payment for this study.

Next

Part 1: Additional instructions

In Part 1 of the study, we will show you **6 different, short stories**.

Each story will be shown on a different page, and each story is approximately eight sentences long, written in English, and intended for a **teenage and young adult audience** (approximately 15 to 24 years of age).

For each story, we ask that you to read it carefully and answer a series of questions about it.

The 6 short stories are about three different topics:

- 2 short stories are about an adventure on the open seas.
- 2 short stories are about an adventure in the jungle.
- 2 short stories are about an adventure on a different planet.

Stories that are about the same topic will be shown one after the another. The order in which you will see the stories and topics is as shown in the list above.

Next

Story (1 of 6)

Instructions: After reading this story, we will ask you a series of questions. The story will remain on the screen after you press continue. Note the story is intended for a **teenage and young adult audience** (approximately 15 to 24 years of age).

Here is the story you are reviewing:

Topic: Write a short story about an adventure on a different planet

Callum and his sister Beth had travelled to many planets with their mother as part of an exploratory mission. There were twelve other teenagers on the ship and they had grown up with each other, experiencing the thrill and dangers of space travel together. As they got older their parents allowed them to take part more in their work. The first time they actually got to do this was on a previously unknown planet called Xephyr . The aliens living on Xephyr were curious to meet the crew and their families and Callum and Beth found that their teenage equivalent on Xephyr were not that different from themselves and they enjoyed making new friends . However, the next uncharted planet was very different...

Please read the story carefully and then advance to the next page.

(You will need to wait at least 10s before you can click the Next button)

Next

Story (1 of 6) Review

Here is the story you are reviewing:

Topic: Write a short story about an adventure on a different planet

Callum and his sister Beth had travelled to many planets with their mother as part of an exploratory mission. There were twelve other teenagers on the ship and they had grown up with each other, experiencing the thrill and dangers of space travel together. As they got older their parents allowed them to take part more in their work. The first time they actually got to do this was on a previously unknown planet called Xephyr . The aliens living on Xephyr were curious to meet the crew and their families and Callum and Beth found that their teenage equivalent on Xephyr were not that different from themselves and they enjoyed making new friends . However, the next uncharted planet was very different...

Please indicate you how much you agree with the following statements on the following scale: 1=Not at all, 5=Moderately, 9=Extremely:

	Not at all 1	2	3	4	5	6	7	8	Extremely 9
**This story is funny.**	○	○	○	○	○	○	○	○	○
**This story has changed what I expect of future stories I will read.**	○	○	○	○	○	○	○	○	○
**This story is boring.**	○	○	○	○	○	○	○	○	○
**This story is well written.**	○	○	○	○	○	○	○	○	○
**I enjoyed reading this story.**	○	○	○	○	○	○	○	○	○
**This story has a surprising twist.**	○	○	○	○	○	○	○	○	○

Next

Story (1 of 6) Review

Here is the story you are reviewing:

Topic: Write a short story about an adventure on a different planet

Callum and his sister Beth had travelled to many planets with their mother as part of an exploratory mission. There were twelve other teenagers on the ship and they had grown up with each other, experiencing the thrill and dangers of space travel together. As they got older their parents allowed them to take part more in their work. The first time they actually got to do this was on a previously unknown planet called Xephyr . The aliens living on Xephyr were curious to meet the crew and their families and Callum and Beth found that their teenage equivalent on Xephyr were not that different from themselves and they enjoyed making new friends . However, the next uncharted planet was very different...

	Not at all 1	2	3	4	5	6	7	8	Extremely 9
**How novel do you think the story is?**	○	○	○	○	○	○	○	○	○
**How original do you think the story is?**	○	○	○	○	○	○	○	○	○
**How rare (e.g. unusual) do you think the story is?**	○	○	○	○	○	○	○	○	○

Next

Story (1 of 6) Review

Here is the story you are reviewing:

Topic: Write a short story about an adventure on a different planet

> Callum and his sister Beth had travelled to many planets with their mother as part of an exploratory mission. There were twelve other teenagers on the ship and they had grown up with each other, experiencing the thrill and dangers of space travel together. As they got older their parents allowed them to take part more in their work. The first time they actually got to do this was on a previously unknown planet called Xephyr . The aliens living on Xephyr were curious to meet the crew and their families and Callum and Beth found that their teenage equivalent on Xephyr were not that different from themselves and they enjoyed making new friends . However, the next uncharted planet was very different...

	Not at all 1	2	3	4	5	6	7	8	Extremely 9
**How appropriate do you think the story is for the intended audience?**	○	○	○	○	○	○	○	○	○
**How feasible to do you think the story is to be developed into a complete book?**	○	○	○	○	○	○	○	○	○
**How likely do you think would it be that the story is turned into a complete book if a publisher read it and hired a professional author to expand on the idea?**	○	○	○	○	○	○	○	○	○

Next

Part 2: Additional instructions

Thank you for completing Part 1.

In Part 2 of the study, we will show you again the **same 6 short stories.**

This time you will be asked **different questions** than before.

After that, you will answer a short follow-up survey and demographic questions.

Next

Story (1 of 6)

Here is the story you are reviewing:

Topic: Write a short story about an adventure on a different planet

Callum and his sister Beth had travelled to many planets with their mother as part of an exploratory mission. There were twelve other teenagers on the ship and they had grown up with each other, experiencing the thrill and dangers of space travel together. As they got older their parents allowed them to take part more in their work. The first time they actually got to do this was on a previously unknown planet called Xephyr . The aliens living on Xephyr were curious to meet the crew and their families and Callum and Beth found that their teenage equivalent on Xephyr were not that different from themselves and they enjoyed making new friends . However, the next uncharted planet was very different...

Please indicate the extent (if any) to which you think this story was based on inputs from an AI tool (e.g. ChatGPT or similar generative AI tool):

0%

0% 25% 50% 75% 100%

Next

Example of a story from the *Human Only* condition: the writer said they did not use an outside AI tool

Story (1 of 6) Review

Here is the story you are reviewing:

Topic: Write a short story about an adventure on a different planet

Many years into the future, a new planet was discovered. The planet was very far away, and not many people knew of its existence. When the first colonists started to arrive there, it was still very wild and dangerous. Despite the danger, many people were drawn to there in pursuit of wealth and adventure. Many others were trying to escape into the unknown wilderness. The rapid advancement of technology had improved many aspects of society on paper, but left some people disillusioned with their lives. These people wanted to return to a more primitive and meaningful way of life. The newly discovered remote planet provided many with the opportunity.

The author of this story was **NOT** provided with **access to AI assistance**. To the best of our knowledge, the author **did not** consult any other AI tool. In short, the author **wrote this story without any input from AI**.

	Not at all 1	2	3	4	5	6	7	8	Extremely 9
**To what extent do you think the story reflects the author's own ideas?**	○	○	○	○	○	○	○	○	○
**To what extent does the author have an "ownership" claim to the final story?**	○	○	○	○	○	○	○	○	○

Next

Example of a story from the *Human Only* condition: the writer said they did use an outside AI tool

Story (3 of 6) Review

Here is the story you are reviewing:

Topic: Write a short story about an adventure on the open seas

....

The author of this story was **NOT** provided with **access to AI assistance**. But the author indicated that **they consulted another AI tool**. In short, the author may have **written the story with input from AI**.

	Not at all<br>1	2	3	4	5	6	7	8	Extremely<br>9
**To what extent do you think the story reflects the author's own ideas?**	○	○	○	○	○	○	○	○	○
**To what extent does the author have an "ownership" claim to the final story?**	○	○	○	○	○	○	○	○	○

Next

Example of a story from the *Human with 1 or 5 AI idea(s)* condition but the author did not access the GenAI idea

Story (2 of 6) Review

Here is the story you are reviewing:

Topic: Write a short story about an adventure on a different planet

....

The author of this story was provided with **access to AI assistance**, which could come up with a starting point for their story. The author **did NOT** choose to make use of the AI assistance. To the best of our knowledge, the author **did not** consult any other AI tool. In short, the author **wrote this story without any input from AI**.

	Not at all 1	2	3	4	5	6	7	8	Extremely 9
**To what extent do you think the story reflects the author's own ideas?**	○	○	○	○	○	○	○	○	○
**To what extent does the author have an "ownership" claim to the final story?**	○	○	○	○	○	○	○	○	○

Next

Example of a story from the *Human with 1 GenAI idea* condition: the writer requested a GenAI idea

Story (3 of 6) Review

Here is the story you are reviewing:

Topic: Write a short story about an adventure in the jungle

Within the heart of an ancient abandoned jungle, an explorer called Robert was exploring this jungle which once had a many tribes on it. While exploring this jungle he found what was left of some of these tribes, abandoned huts, old fire places, and bones. He didn't know what could of caused all of the tribes to die out. As he was walking, Robert felt a strange presence in the jungle, he saw some footsteps going deep in the jungle.
He followed the footsteps until he found a temple which was build inside a mountain, He walked inside of the temple he saw 3 paths going into different directions, each of the paths were lit up by torches, he knew what that meant. Robert took the path that went straight, but said that these paths went extremely deep into the mountain, until he heard some sounds, that were of the remaining people of this jungle, he thought to himself that they might be the people that killed all the other tribes. Robert watched from away as the people were having their meal, when he stumbled and they heard him and turned around, He saw what looked to be the leader of the tribe walking towards him. Robert didn't try to run away but question the people to understand what was happening, the leader said that they didn't know about the other tribes being dead, and they were very friendly with him, they invited him to have his meal with them, and offered him to stay the night, he accepted the offer, he went to sleep, and after that the leader of the tribe killed him in his sleep.

The author of this story was provided with **access to AI assistance**, which could come up with a starting point for their story. The author **chose to make use** of the AI assistance and the AI assistance provided the following prompt(s) to the author:

AI suggestion 1
While exploring a mysterious ancient temple deep in the heart of the jungle, a group of friends accidentally awakens a long-forgotten entity that guards the temple. Now they must race against time to decrypt hidden clues and solve cryptic puzzles to deactivate the ancient curse before it destroys everything in its path. Friendships are tested, secrets are revealed, and through their daring adventure, the group discovers hidden strengths within themselves that give them the courage to save their lives and secure the temple's treasures.

In short, the author may have **written the story with input from AI**.

	Not at all 1	2	3	4	5	6	7	8	Extremely 9
**To what extent do you think the story reflects the author's own ideas?**	○	○	○	○	○	○	○	○	○
**To what extent does the author have an "ownership" claim to the final story?**	○	○	○	○	○	○	○	○	○

If this story were published and sold tomorrow, how much of the story's profit do you believe should belong to **the author** versus **the creators of the generative AI tool** that may have provided the starting point for the story?

0%

0% 25% 50% 75% 100%

Next

Example of a story from the *Human with 5 GenAI ideas* condition: the writer requested five GenAI ideas

Story (1 of 6) Review

Here is the story you are reviewing:

Topic: Write a short story about an adventure on a different planet

Callum and his sister Beth had travelled to many planets with their mother as part of an exploratory mission. There were twelve other teenagers on the ship and they had grown up with each other, experiencing the thrill and dangers of space travel together. As they got older their parents allowed them to take part more in their work. The first time they actually got to do this was on a previously unknown planet called Xephyr . The aliens living on Xephyr were curious to meet the crew and their families and Callum and Beth found that their teenage equivalent on Xephyr were not that different from themselves and they enjoyed making new friends . However, the next uncharted planet was very different...

The author of this story was provided with **access to AI assistance**, which could come up with a starting point for their story. The author **chose to make use** of the AI assistance and the AI assistance provided the following prompt(s) to the author:

AI suggestion 1
Three friends, Abigail, Elliot, and Maddox, accidentally stumble upon an intergalactic portal, landing on the mysterious Planet Zephyr. Battling harsh alien terrain, inhospitable creatures and limited oxygen supplies, they embark on a daring mission to locate a legendary power source reportedly capable of returning them back to Earth. The trio's bravery, intelligence, and unyielding spirit are put to the ultimate test, in a high-stakes, high-adrenaline off-world adventure unlike anything they'd ever imagined.

AI suggestion 2
When a group of young astronauts land on the uncharted planet Xerxes V, they discover a vibrant ecosystem filled with sentient plant life. Challenged by the complex society of these plants and a rapidly dwindling oxygen supply, the group must harness their courage, intellect and adaptability to understand the alien civilization and work with them to survive. The unexpected unity of human and plant life on Xerxes V results in an astonishing adventure depicting interstellar diplomacy and the overwhelming power of cooperation.

AI suggestion 3
Three earthlings inadvertently stumble upon a portal leading to an exotic yet perilous planet named Zephyros. Their brave adventures are filled with encounters of strange, alien species, near-death experiences due to the planet's unpredictable weather, and the resolution of an age-old Zephyri conflict. Eventually, they return to Earth with tales of their remarkable interstellar adventures and hitherto unknown insights about the cosmos.

AI suggestion 4
In quest of finding signs of life beyond Earth, a group of astronauts embark on a thrilling journey to Prolion-6, an unexplored planet in a distant galaxy. They encounter a complex ecosystem of intelligent, peaceful alien species and discover ancient technologies far advanced than humanity's. Their adventure reveals a profound interstellar culture with cosmic potential, prompting both awe and existential thoughts.

AI suggestion 5
Three human explorers journey to an uncharted planet, Epsilon Prime, in search of precious minerals. They encounter alien life that communicates through color-changing patterns on their skin, causing initial conflict due to misunderstood intentions. Through cooperation, both parties learn to understand each other, leading to a newfound interspecies alliance and the successful completion of the explorer's mission.

In short, the author may have **written the story with input from AI**.

	Not at all 1	2	3	4	5	6	7	8	Extremely 9
**To what extent do you think the story reflects the author's own ideas?**	○	○	○	○	○	○	○	○	○
**To what extent does the author have an "ownership" claim to the final story?**	○	○	○	○	○	○	○	○	○

If this story were published and sold tomorrow, how much of the story's profit do you believe should belong to **the author** versus **the creators of the generative AI tool** that may have provided the starting point for the story?

0%

0% 25% 50% 75% 100%

Next

AI Overview

Please indicate you how much you agree with the following statements on the following scale: 1=Not at all, 5=Moderately, 9=Extremely:

	Not at all 1	2	3	4	5	6	7	8	Extremely 9
**If a human creator (author) uses AI in part of the writing of a story, the AI-generated content should be accessible alongside the final story.**	○	○	○	○	○	○	○	○	○
**It is ethically acceptable to use AI to come up with an initial idea for a story.**	○	○	○	○	○	○	○	○	○
**If AI is used in any part of the writing of a story, the creators of the content on which the AI output was based on should be compensated.**	○	○	○	○	○	○	○	○	○
**If AI is used in any part of the writing of a story, the final story no longer counts as a "creative act".**	○	○	○	○	○	○	○	○	○
**Relying on the use of AI to write a new story is unethical.**	○	○	○	○	○	○	○	○	○
**It is ethically acceptable to use AI to write and publicly disseminate an entire story without acknowledging the use of AI.**	○	○	○	○	○	○	○	○	○

Next

Follow-up survey

	Not at all<br>1	2	3	4	5	6	7	8	Extremely<br>9
**How creative do you consider yourself?**	○	○	○	○	○	○	○	○	○
**How much creativity is required in your job?**	○	○	○	○	○	○	○	○	○
**How comfortable are you with new technologies?**	○	○	○	○	○	○	○	○	○
**How much (if at all) have you previously engaged with AI or similar technologies?**	○	○	○	○	○	○	○	○	○

Have you used any of the following AI tools in the past? (Check all that apply)

- ☐ None
- ☐ ChatGPT
- ☐ Dall-E
- ☐ OpenAI's playground (e.g. DaVinci, Currie, Ada)
- ☐ Stable Diffusion
- ☐ NightCafe
- ☐ Jasper
- ☐ Microsoft Bing Chat
- ☐ Google Bard
- ☐ You.com
- ☐ Midjourney
- ☐ Other

Ai Tools Other Name

____

Have you used any of the following categories of AI tools in the past? (Check all that apply)

- ☐ None
- ☐ Text
- ☐ Image
- ☐ Audio
- ☐ Music
- ☐ Video

Next

Demographics

What gender do you identify with?

- ○ Female
- ○ Male
- ○ Prefer not to say
- ○ Other (please specify below)

Other gender

[ ]

What is your current age? (enter a number of years)

[ ]

What is your highest level of education?

- ○ Less than A levels
- ○ Vocational training
- ○ A levels
- ○ Undergraduate degree
- ○ Postgraduate Master's degree
- ○ Professional degree (e.g. MBA, JD)
- ○ Doctorate

What is your current employment status?

- ○ Employed full time
- ○ Employed part time
- ○ Unemployed looking for work
- ○ Unemployed not looking for work
- ○ Retired
- ○ Student
- ○ Disabled

What is your current job title?

[ ]

What is your current annual income?

- ○ Less than £10,000
- ○ £10,000-£24,999
- ○ £25,000-£49,999
- ○ £50,000-£74,999
- ○ £75,000-£99,999
- ○ £100,000-£124,999
- ○ £125,000-£149,999
- ○ More than £150,00

Do you have any additional comments about this survey?

[ ]

Next

Section 9. Pre-registered analysis (AsPredicted #136723)

ASPREDICTED

Wharton CREDIBILITY LAB
UNIVERSITY of PENNSYLVANIA

CONFIDENTIAL - FOR PEER-REVIEW ONLY

Generative Artificial Intelligence and Creative Production (#136723)

Created: 06/26/2023 02:06 AM (PT)

This is an anonymized copy (without author names) of the pre-registration. It was created by the author(s) to use during peer-review.
A non-anonymized version (containing author names) should be made available by the authors when the work it supports is made public.

1) Have any data been collected for this study already?

No, no data have been collected for this study yet.

2) What's the main question being asked or hypothesis being tested in this study?

How does the availability of generative AI to assist with a creative task (i.e. writing a short story) affect the (self-)evaluation of the creative output by creators and third-party evaluators?

3) Describe the key dependent variable(s) specifying how they will be measured.

We are interested in assessing the following dependent variables, which will be measured for both creators and third-party evaluators:
- Novelty: an index of three questions on a scale of 1 to 9
- Usefulness: an index of three questions on a scale of 1 to 9

We will create aggregate indices for these measures, as well as look at their individual components.

4) How many and which conditions will participants be assigned to?

There are three conditions in the study:
- "Human only" condition where the project creator does not get any AI assistance
- "Hybrid" condition where the creator has the opportunity to access one short prompt for a story idea from OpenAI's ChatGPT API
- "Hybrid+" condition where the creator has the opportunity to access up to five short prompts for a story idea from OpenAI's ChatGPT API

5) Specify exactly which analyses you will conduct to examine the main question/hypothesis.

We will run OLS regressions predicting novelty and usefulness by condition, and run these regressions for both creators and evaluators.

We will run robustness tests for each of these, which will include different econometric specifications and variants (e.g., sub-items, discretization) of the outcome measures.

Note: While the Hybrid and Hybrid+ conditions technically differ in their capabilities (the latter allows for more AI prompts), we plan to combine the two conditions into one joint condition for our main analysis if the main outcome variables in those two conditions are not statistically significant from each other. (We will still report the existence of all three conditions and a results breakdown by all three conditions in the appendix.)

6) Describe exactly how outliers will be defined and handled, and your precise rule(s) for excluding observations.

We will exclude all participants that did not finish the study for analysis purposes. We will also drop respondents in the "Human only" condition that acknowledged that they used generative AI to assist with their responses.

7) How many observations will be collected or what will determine sample size? No need to justify decision, but be precise about exactly how the number will be determined.

For each condition, we will collect n=100 creators per condition who complete the study, for a total of n=300 creators across the three conditions. Then, we will collect n=600 third-party evaluators (each of which evaluates six stories drawn at random from the creator conditions).

8) Anything else you would like to pre-register? (e.g., secondary analyses, variables collected for exploratory purposes, unusual analyses planned?)

We have additional exploratory outcome variables about each story (e.g. enjoyment of the story, how well written, funny or boring the story is, etc.) on a scale from 1 to 9, for which we will study treatment effects similar to our main analysis.

We have collected a number of variables that we will use to look at heterogeneous effects including the creativity of the respondent (through a creativity task prior to the writing task and through self-ratings), their prior experience with generative AI technologies, and demographic information (e.g. gender, education, employment and income).

We will also consider non-linear relationships, by discretizing the outcomes and running linear probability models.
